# A Sustainable Workflow for the Recovery of Glucosylceramides from Apple Pomace

**DOI:** 10.3390/molecules31213807

**Published:** 2026-10-22

**Authors:** Ghislain Fotso Wabo, Samuel Ngure Kariithi, Norbert Arnold, Jörg Ziegler, Bernhard Westermann

**Affiliations:** 1 Department of Bioorganic Chemistry, Leibniz Institute of Plant Biochemistry, Weinberg 3, D-06120 Halle (Saale), Germany; norbert.arnold@ipb-halle.de (N.A.); bernhard.westermann@ipb-halle.de (B.W.); 2 Program Center for Plant Metabolomics and Computational Biochemistry, Leibniz Institute of Plant Biochemistry, Weinberg 3, D-06120 Halle (Saale), Germany; joerg.ziegler@ipb-halle.de; 3 Institute of Chemistry, Martin-Luther-University Halle-Wittenberg, Kurt-Mothes-Strasse 2, D-06120 Halle (Saale), Germany

**Keywords:** Bodensee organic pomace, Saale-Unstrut organic pomace, Saale-Unstrut conventional pomace, sustainable workflow, UHPLC-VIP-HESI-MS/MS, 1D/2D NMR spectroscopy, phytosphingoid-based phytoceramides, total GlcCER content, circular bioeconomy

## Abstract

Apple pomace, a major agro-industrial byproduct of the juice industry, remains an underexploited renewable source of high-value sphingolipids. In this study, we developed an integrated, more sustainable workflow for the enrichment and recovery of phytoceramides from three apple pomace samples differing in geographical origin, cultivation practice, and processing history using chlorinated and *n*-hexane-free solvents. Nine hydroalcoholic solvent systems were evaluated by ultrasound-assisted extraction to balance extraction efficiency and environmental performance. UHPLC-VIP-HESI-MS/MS lipidomics of the phytoceramide-enriched fractions enabled a putative annotation of 64 CER and GlcCER molecular species, with phytosphingoid-based species predominating. Subsequent purification of these fractions by semi-preparative HPLC yielded four major GlcCERs, whose structures were elucidated by high-resolution MS and 1D/2D NMR spectroscopy. Total GlcCER content was 391 mg/Kg of dry pomace for Bodensee organic pomace (E1), 264 mg/Kg, for Saale-Unstrut organic pomace (E2), and 402 mg/Kg for Saale-Unstrut conventional pomace (E3). Overall, the workflow integrates green extraction, sustainable downstream processing, and advanced molecular characterization, demonstrating the potential of apple pomace for high-value sphingolipid valorization within a circular bioeconomy. Furthermore, this streamlined three-step process demonstrates feasibility at kilogram scale and provides a practical basis for further scale-up studies toward potential industrial application.

## 1. Introduction

Ceramides (CERs) are an essential class of sphingolipids present in the Stratum corneum, the outermost layer of the epidermis, where they play a fundamental role in maintaining skin barrier integrity and homeostasis [1]. The Stratum corneum consists of corneocytes embedded within a highly organized intercellular lipid matrix primarily composed of Ceramides, cholesterol, and free fatty acids in an approximately equimolar ratio [2]. CERs are composed of a sphingoid base linked to a fatty acid and serve as essential components of the epidermal lipid matrix, where they regulate water retention, protect against environmental stressors, and contribute to skin homeostasis [3]. Beyond their structural role, CERs act as bioactive signaling molecules involved in diverse cellular processes, including apoptosis, cell differentiation, inflammation, and stress responses. Advances in lipidomics and molecular biology have expanded our understanding of ceramide subclasses, their enzymatic regulation, and their potential as therapeutic targets. Recently, α-galactosylceramides (α-GalCERs) have gained attention as vaccine adjuvants [4]. Dysregulation of ceramide metabolism has been implicated in various pathological conditions, including atopic dermatitis, psoriasis, metabolic syndrome, insulin resistance, and neurodegenerative diseases [3]. Ceramides’ role in skin homeostasis makes them an important ingredient widely used in the cosmetic industry in the formulation of skin care products. Ceramides used in the cosmetic industry are mostly synthetic or derived from animal sources such as bovine brain, porcine skin, and hen eggs. However, the use of bovine-derived materials has raised safety concerns because of the potential transmission of prions responsible for bovine spongiform encephalopathy (BSE) and its associated human variant, Creutzfeldt–Jakob disease [5]. As an alternative, CERs and GlcCERs isolated from plants such as apple pomace provide a safer source of skin-similar CERs [1,6]. Worldwide, it is estimated that up to 5 million metric tons of agro-industrial by-products, such as apple pomace, are generated annually [7]. Previous phytochemical studies have established apple pomace as a rich source of valuable secondary metabolites, including flavonoids such as quercetin, phloretin, and phloridzin, hydroxycinnamic acids such as chlorogenic and caffeic acids, and pentacyclic triterpenoids, with ursolic acid reported as the predominant triterpenoid in apple pomace. Pectic oligosaccharides, including galacturonyl oligosaccharides, and glucosylceramides further contribute to its potential as a valuable feedstock for the recovery of high-value compounds within circular bioeconomy strategies [8,9,10,11,12,13]. However, the isolation of GlcCERs from such complex plant matrices remains analytically challenging, particularly when considering environmental sustainability constraints. Historically, lipid extraction protocols have relied on conventional solvent systems such as chloroform–methanol mixtures, as established by the Folch and Bligh–Dyer methods [14,15]. While highly efficient, these approaches involve chlorinated solvents that present significant environmental and toxicological concerns. Furthermore, petroleum-derived hydrocarbons such as *n*-hexane are still widely employed during liquid-liquid partitioning and chromatographic fractionation despite concerns related to neurotoxicity, volatile organic compound (VOC) emissions, and environmental sustainability [16,17,18,19,20]. Despite growing awareness of green chemistry principles, these methods remain widely used due to their robustness and reproducibility in lipidomics workflows [21]. In response to these limitations, considerable efforts have been directed toward the development of green extraction technologies. Recent reviews highlight the emergence of alternative approaches such as ultrasound-assisted extraction (UAE), microwave-assisted extraction (MAE), pressurized liquid extraction (PLE), enzyme-assisted extraction (EAE), and supercritical fluid extraction (SFE), which significantly reduce solvent consumption, energy input, and processing time while improving extraction efficiency [22,23]. Notably, a recent comprehensive review emphasizes that these green technologies can increase extraction yields by up to threefold while reducing energy consumption by approximately 40% [24]. However, despite these advances, the transition toward fully green workflows remains incomplete. Most studies focus primarily on the extraction step, whereas downstream processes such as fractionation and purification continue to rely on conventional chromatographic techniques using non-green solvents [21,25]. To date, most efforts have focused on developing greener extraction methods, whereas sustainable downstream fractionation and purification strategies for plant sphingolipids remain largely unexplored. Consequently, no integrated workflow eliminating both chlorinated solvents and *n*-hexane throughout extraction, fractionation, and purification has been reported for the recovery of CERs and GlcCERs from apple pomace. To the best of our knowledge, this study is among the first to report a more sustainable and scalability-oriented workflow for the extraction, fractionation, purification, and isolation of plant glucosylceramides from apple pomace, while preserving high recovery yields and enabling the structural characterization of the major molecular species. Beyond improving process sustainability, the proposed workflow demonstrates that hazardous solvents can be eliminated without compromising extraction efficiency, thereby providing a practical strategy for the sustainable valorization of apple pomace within a circular bioeconomy framework.

## 2. Results and Discussion

### 2.1. Determination of the Moisture Content of the Three Apple Residues

Because the moisture content of plant materials directly influences extraction efficiency, solvent composition, yield calculations, and storage stability, it was determined for the three apple pomace samples prior to extraction (Figure S1, Supplementary Data). The measured values were 6.83 ± 0.11% for the organic Bodensee sample (E1), 6.21 ± 0.04% for the organic Saale-Unstrut sample (E2), and 4.00 ± 0.09% for the conventional Saale-Unstrut sample (E3). The results indicate that organic apple pomace samples exhibited higher moisture contents than the conventional sample, irrespective of the cultivation area or extraction process. Furthermore, all samples presented moisture levels below 10%, comparable to those available in the literature for apple pomace [11], suggesting good storage stability and a reduced susceptibility to microbial infestation and degradation [26]. The measured moisture contents were considered during both small- and large-scale extraction procedures to adjust the water proportion accordingly. These parameters should also be considered in potential industrial-scale applications involving larger quantities of apple pomace.

### 2.2. Sustainable Selection of the Optimal Extraction Method

To identify the most suitable solvent combining extraction efficiency, safety, and sustainability for large-scale extraction, 1.0 g of each apple pomace sample (E1, E2, and E3) was initially subjected to ten ultrasound-assisted extraction (UAE) treatments. In addition to the conventional ternary solvent system isopropanol–*n*-hexane–water (55:20:25, *v*/*v*/*v*) previously reported by Reisberg et al. and Adem et al. [27,28], nine hydroalcoholic solvent mixtures free of the class 2 toxic solvent *n*-hexane were evaluated (Section 3.4.1). It should be noted that the comparison between methanol and other extraction systems was performed under different temperature conditions. Methanol–water extractions were conducted at 50 °C, whereas ethanol–water extractions were conducted at 70 °C. This difference was introduced because substantial evaporation of methanol occurred at 70 °C under the ultrasonic extraction conditions, making it difficult to maintain a constant solvent composition and extraction volume. The hydroalcoholic solvent systems were deliberately selected according to Green Chemistry principles and internationally recognized solvent selection guides (CHEM21 and the Green Solvent Selection Guides), which recommend replacing hazardous petroleum-derived solvents with safer alternatives having lower toxicity, improved environmental profiles, and greater potential for sustainable industrial implementation without compromising extraction performance [15,19]. *n*-Hexane is a petroleum-derived volatile organic compound (VOC) classified as a Class 2 solvent under the ICH Q3C guideline [16] because of its neurotoxicity and the need to limit residual solvent levels in pharmaceutical and related products. Moreover, its high volatility contributes to atmospheric VOC emissions, while its fossil origin is inconsistent with current strategies aimed at reducing the environmental footprint of extraction processes. The replacement of *n*-hexane was intentionally undertaken to develop a safer and more sustainable extraction protocol. Unlike conventional lipid extraction methods relying on petroleum-derived solvents, the hydroalcoholic systems evaluated in this study are composed of solvents with lower toxicity and improved environmental profiles, in accordance with current green extraction principles [20,29].

### 2.3. Quantitative Analysis of the Extracts Yields

**Figure 1:**
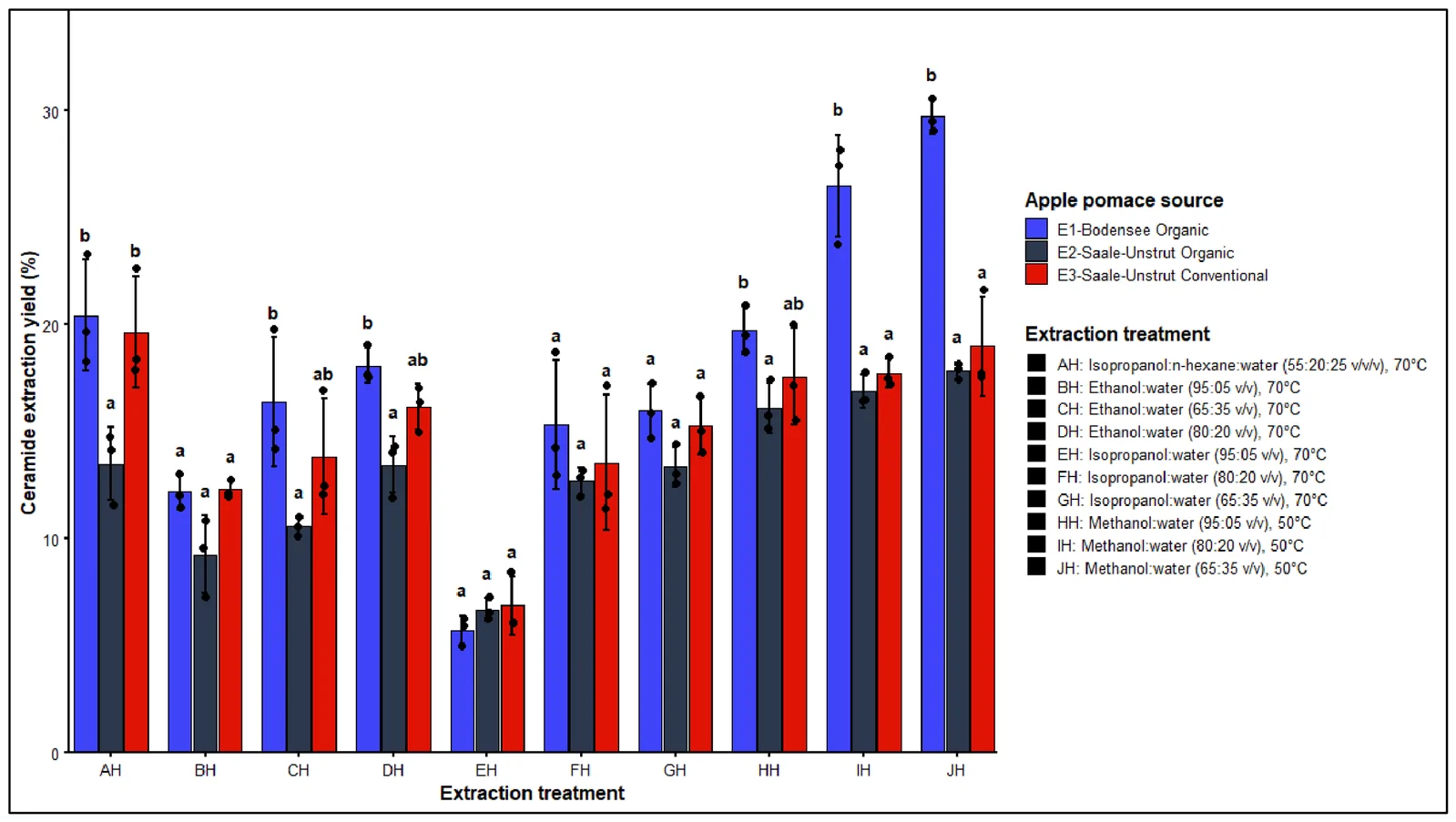
Comparative evaluation of crude extract yield from three apple pomace sources using different extraction treatments. Crude extract yield was determined for E1 (Bodensee Organic), E2 (Saale-Unstrut Organic), and E3 (Saale-Unstrut Conventional) and expressed as a percentage of the initial sample mass (1 g). Bars represent mean ± SD, with individual data points shown (*n* = 3 different extractions from same batch of apple pomace). Different lowercase letters indicate significant differences among apple pomace sources within each extraction treatment, as determined by two-way ANOVA followed by Tukey’s HSD test (*p* < 0.05). Note that the letters do not indicate statistical comparisons between extraction treatments.

The extraction yields obtained (Figure 1) highlighted the relatively strong influence of solvent composition on extraction efficiency, with methanol–water mixtures providing the highest yields (16.1–29.7%) even at lower temperatures. Ethanol-based solvent systems exhibited slightly lower extraction yields (9.3–18.1%) but remained attractive alternatives due to their low toxicity and broad regulatory acceptance as green solvents [20]. In contrast, isopropanol-based systems showed the lowest extraction efficiencies (5.7–16.0%), likely reflecting their lower polarity relative to methanol and ethanol. The ternary solvent system (AH), combining *n*-hexane with polar solvents, produced intermediate and more variable yields (13.5–20.4%), which may be explained by phase partitioning effects and reduced selectivity toward amphiphilic lipids.

A pronounced effect of the raw material processing history on extraction yield was also observed, with sample E1 consistently exhibiting higher extraction yields than E2 and E3. Since E1 was obtained after a single extraction process, it likely retained a greater proportion of secondary metabolites. In contrast, E2 and E3 underwent additional industrial treatments, including enzymatic degradation followed by a second extraction step, which likely contributed to the reduced extraction yields. Enzymatic treatments commonly applied during juice processing promote cell wall degradation and facilitate the release, and potential loss, of intracellular compounds [30,31].

### 2.4. Qualitative Analysis of the Extracts by UHPLC-VIP-HESI-MS/MS

**Figure 2:**
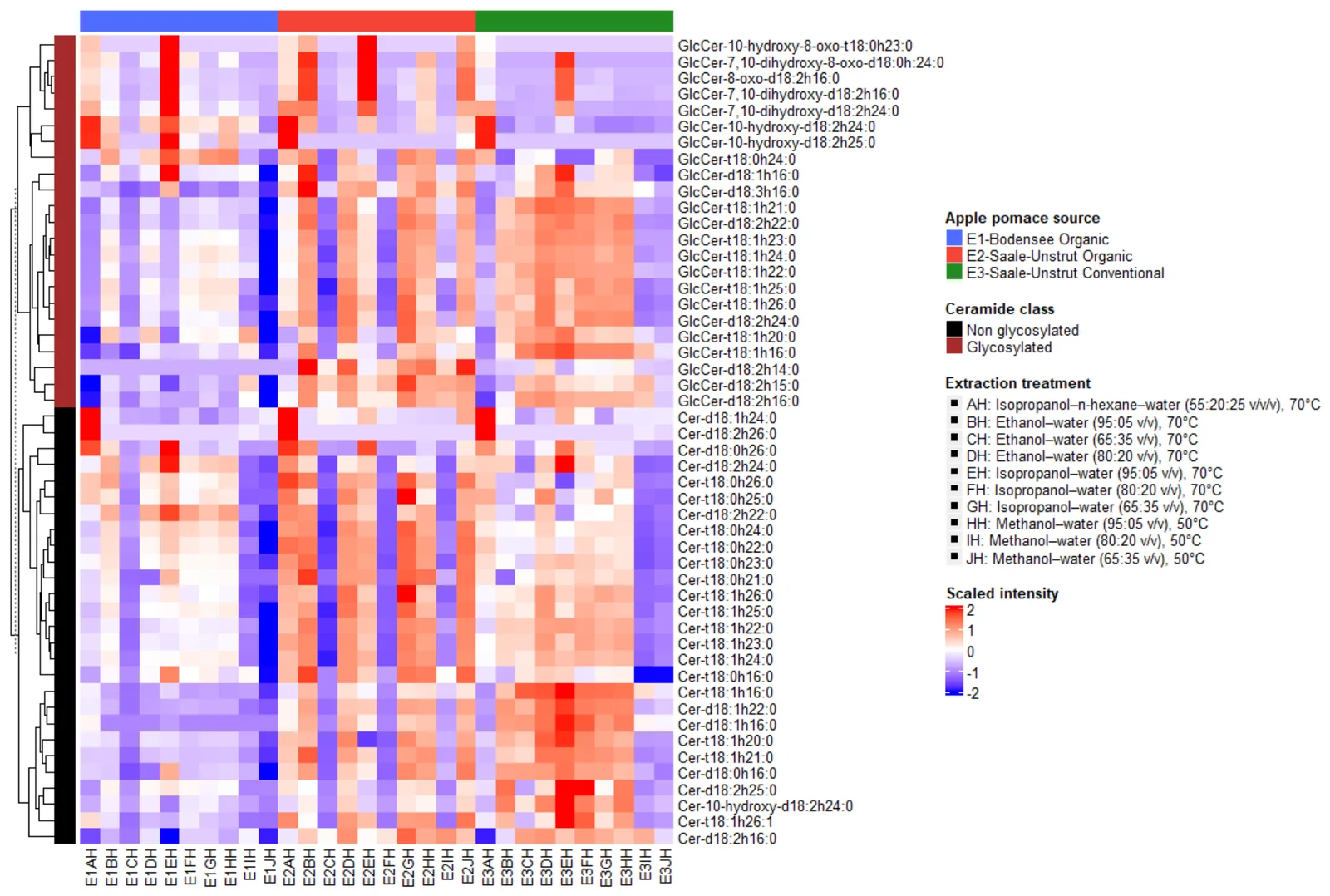
Heatmap showing the relative abundance of CERs and GlcCERs identified in apple pomace extracts obtained using different extraction treatments. Rows represent individual CERs (Black) or GlcCER (maroon) species and columns represent biological samples (E1, Bodensee Organic; E2, Saale-Unstrut Organic; E3, Saale-Unstrut Conventional), under different extraction treatments (AH–JH). Peak intensities were normalized to the peak intensity of the internal standard, deuterated phosphatidylethanolamine (d_7_ PE 15:0-18:1), and subsequently log-transformed. For each sample, values from five technical replicate injections were averaged before heatmap visualization (*n* = 5 technical replicate injections per sample). The color scale represents log-transformed, internal standard-normalized peak intensities, with red indicating high and blue indicating low relative abundance.

To assess the quality of the thirty extracts obtained from the small-scale extraction of the three apple pomace samples (E1, E2, and E3) using the ten different extraction treatments, phytoceramides were putatively annotated in each sample by UHPLC-VIP-HESI-MS/MS. A heatmap was then generated by comparing the relative abundances of the annotated phytoceramides across all samples (Figure 2). These relative signal intensities provided a comparative overview of the detected phytoceramide profiles but should not be interpreted as direct measures of extraction yields or absolute phytoceramide recovery, as they may also be influenced by factors such as matrix effects and ionization efficiency. In a previous study, Reisberg et al. (2017) [27] annotated a total of 45 phytoceramides from apple pomace, wheat germ, and coffee husks using the ternary isopropanol–*n*-hexane–water (AH) extraction method. In the present study, 50 phytoceramides were putatively annotated from apple pomace, demonstrating that the alcohol-based extraction treatments achieved extraction performance comparable to that of the reference. However, the number of annotated compounds alone does not establish equivalent quantitative recovery relative to the reference method.

Based on the heatmap (Figure 2), the reference extraction treatment AH generally showed high relative abundances for CER species across the three apple pomace samples, while the corresponding pattern for GlcCER was less pronounced. Ethanol–water hydro-alcoholic mixtures (BH and DH) showed high relative abundances for both CER and GlcCER across the three samples, whereas extraction treatment CH showed lower relative abundances, especially in E1 and E2. In comparison to the reference treatment method (AH), isopropanol–water hydro-alcoholic mixtures displayed variable relative intensities, ranging from moderate to relatively high for both CER and GlcCER species across all the study sites. However, due to their low quantitative crude extract yields, they were considered unsuitable for this study. The low yields refer to the total mass of extract obtained and do not refer to low or high phytoceramide content. Notably, despite the high quantitative yields displayed by methanol-based extractions, annotated phytoceramides exhibited low relative abundances for both CERs and GlcCERs across the three samples, except for the HH methanol-based extraction, which showed relatively higher relative abundances than the reference method AH (Figure 2). Furthermore, the overall similarity between IH (80:20 methanol–water) and JH (65:35 methanol–water) indicated that reducing the methanol concentration had little influence on the CER and GlcCER profile under the extraction conditions employed. Overall, the ethanol–water treatments consistently exhibited relatively high relative abundances for a larger number of CER and GlcCER species than the other treatments (Figure 2). These findings indicate favorable qualitative phytoceramide profiles but should not be interpreted as evidence of proportionally greater absolute recovery without quantitative validation.

Taking into consideration the obtained yields, relative abundances, solvent safety, sustainability, and selective recovery of CERs and GlcCERs, an ethanol–water (80:20, *v*/*v*) mixture was selected as the preferred extraction treatment for subsequent analysis. This treatment represents a compromise between extract yield, the observed phytoceramide profile, and environmental considerations rather than a definitive demonstration of the highest absolute phytoceramide recovery. From a Green Chemistry perspective, this solvent system offers a potentially more sustainable alternative to the conventional isopropanol–*n*-hexane–water extraction system. Although both ethanol and isopropanol are commonly used as solvents, ethanol is considered generally favorable in terms of its potential renewable origin, biodegradability, and lower toxicity. Replacing the fossil-resource-derived n-hexane and isopropanol with an ethanol-water solvent provides us with an alternative to avoid the petroleum-derived solvents associated with significant health and environmental concerns [17,19,20]. Therefore, compared to the reference extraction method AH, ethanol–water (80:20, *v*/*v*) was selected as the preferred method based on the combined crude extract yield, phytoceramide relative abundances, and sustainability considerations.

### 2.5. Sustainable Fractionation of the Crude Extracts

Apple pomace crude extracts constitute highly complex phytochemical matrices in which phytoceramides and glucosylceramides are accompanied by a wide range of primary and secondary metabolites, including sugars, organic acids, amino acids, phenolic compounds, flavonoids, triterpenoids, fatty acids, neutral lipids, phospholipids, pigments, and other membrane-associated lipids [30,32]. Therefore, fractionation represents a critical step for reducing matrix complexity, removing interfering compounds, enriching sphingolipid-containing fractions, and facilitating the subsequent purification and structural characterization of GlcCERs. Despite recent efforts to develop greener extraction and purification workflows of natural products, chlorinated solvents remain widely employed in lipid and sphingolipid isolation protocols for liquid–liquid partitioning, silica gel chromatography, and Sephadex LH-20 because of their excellent lipid-solubilizing capacity, low hydrogen-bonding ability, and good miscibility with polar organic solvents such as methanol. Similarly, petroleum-derived hydrocarbons such as *n*-hexane are still extensively used during liquid–liquid extraction and chromatographic fractionation because of their strong affinity for neutral lipids and their ability to improve phase separation [20,29]. However, both solvents are classified as hazardous owing to their toxicity and adverse environmental impacts [18]. Chlorinated solvents such as chloroform and dichloromethane are of particular concern because of their high human toxicity, persistence in the environment, difficulties associated with waste treatment, and contribution to hazardous solvent emissions. Moreover, dichloromethane is classified as a suspected carcinogen, while chloroform is recognized as a probable human carcinogen and presents significant risks to both operators and the environment. Together with the neurotoxicity and volatile organic compound (VOC) emissions associated with *n*-hexane [17,18,19,20], these drawbacks have motivated the development of alternative purification strategies based exclusively on safer and non-chlorinated solvents.

The proposed protocol (Scheme S1, Supplementary Data), which combined vacuum liquid chromatography (VLC) with size-exclusion chromatography on Sephadex LH-20 using only ethyl acetate (~3.5 L/Kg of apple pomace) and methanol (~1.5 L/Kg of apple pomace), efficiently removed the major lipophilic constituents, including fatty acids, triterpenoids, sterol derivatives, and flavonoids of the apple pomace crude extracts, yielding a GlcCER-enriched fraction (Figure S2, Supplementary Data). Subsequent precipitation of this fraction with an acetone–methanol (9:1, *v*/*v*) mixture was crucial for removing residual phenolic compounds, thereby producing a highly GlcCER-enriched precipitate reaching 516.0 mg/kg for E1, 368.0 mg/kg for E2, and 473.8 mg/kg for E3 (Table 1). The observed variation indicates that both the origin of the raw material, cultivation practice, and its processing history influence the GlcCER content of apple pomace. Similar variability in plant glycolipid composition has previously been reported as a function of environmental and agronomic factors [33,34]. Overall, these results demonstrate that direct VLC fractionation following extraction, combined with Sephadex LH-20 chromatography and acetone-based precipitation, constitutes a rapid, efficient and more sustainable strategy for recovering GlcCER-enriched fractions from both organically and conventionally produced apple pomaces.

### 2.6. Green Chemistry Assessment of the Fractionation Workflow

The sustainability of the developed fractionation workflow relies on a holistic Green Chemistry approach extending beyond simple solvent substitution. In addition to completely eliminating chlorinated solvents and *n*-hexane traditionally employed for sphingolipid purification, the workflow was designed to minimize solvent consumption, simplify downstream processing, enable solvent recycling, and maintain high GlcCER recoveries. Consequently, the proposed strategy addresses several key principles of Green Chemistry, including the use of safer solvents and auxiliaries, waste prevention, inherently safer chemical processes, and improved resource efficiency [17,19].

Unlike conventional sphingolipid purification protocols relying on repeated liquid-liquid partitioning with chlorinated solvents and petroleum-derived hydrocarbons. By using solvent recycling, the present workflow required only approximately 3.5 L of ethyl acetate, 1.5 L of methanol, and 300 mL of acetone per kilogram of apple pomace to produce the GlcCER-enriched precipitates. This substantial reduction in solvent consumption not only lowers the amount of hazardous waste generated but also decreases solvent handling, storage, transportation, and disposal requirements. Because solvent manufacture and use account for a significant proportion of the environmental burden of chemical processes, solvent minimization represents an essential strategy for improving the overall sustainability of natural product isolation [18,19].

Overall, the developed purification strategy demonstrates that alternative, comparatively more sustainable solvent systems can replace chlorinated solvents and *n*-hexane during downstream sphingolipid processing without compromising GlcCER recovery. The combination of solvent substitution, solvent minimization, solvent recycling, simplified purification, and efficient recovery establishes the proposed workflow as a practical example of Sustainable Chemistry applied to the valorization of renewable apple pomace. These features reduce reliance on chlorinated solvents and *n*-hexane and provide a practical basis for further investigation of the environmental and economic feasibility of GlcCER recovery from agro-industrial residues.

### 2.7. Qualitative Analysis of the Enriched Fractions by UHPLC-VIP-HESI-MS/MS

**Figure 3:**
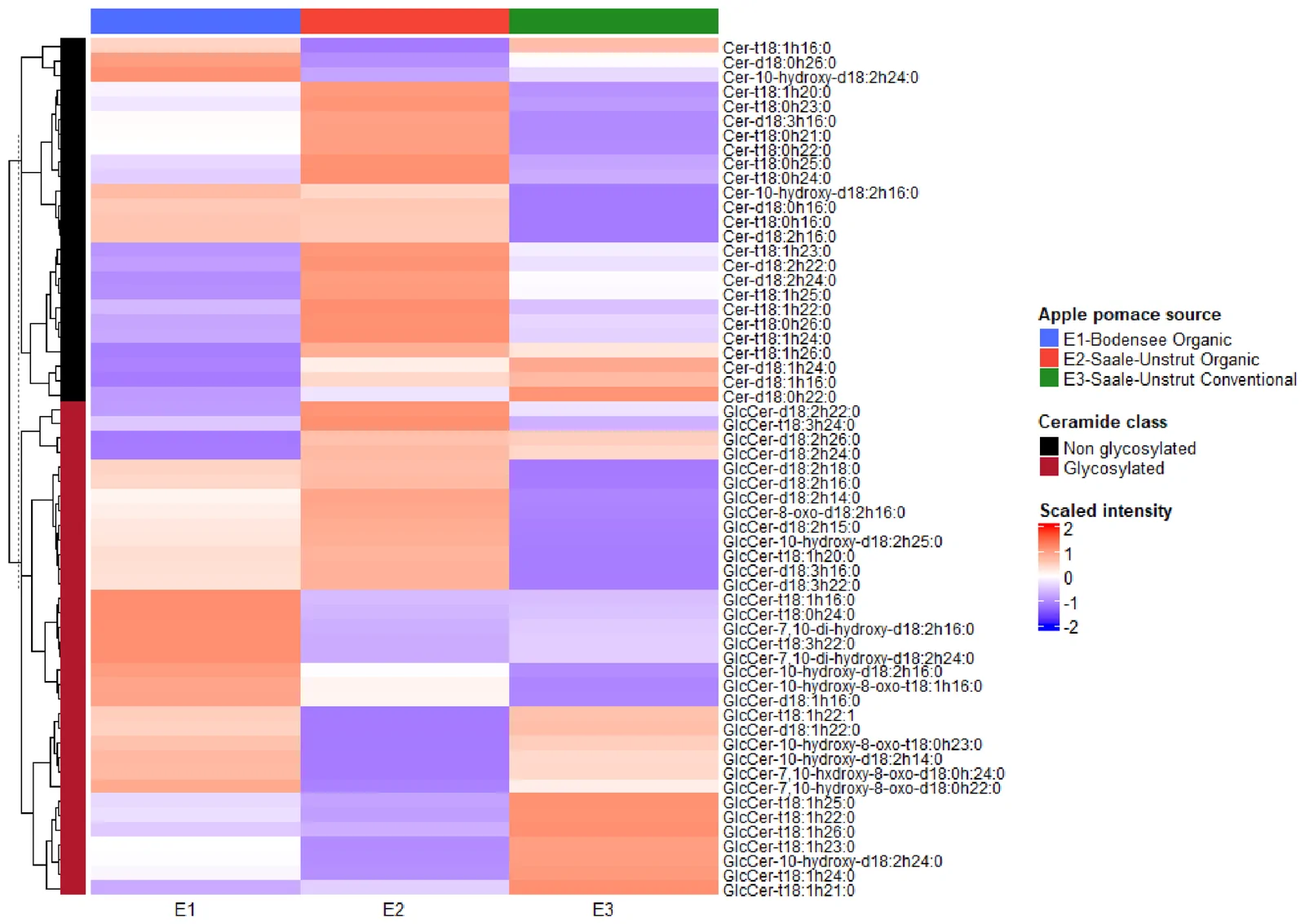
Heatmap showing the relative abundance of CERs and GlcCERs identified in apple pomace-enriched precipitates from three different sources. Rows represent individual CERs (Black) or GlcCER (Maroon) species and columns represent enriched precipitates from three biological samples (E1, Bodensee Organic; E2, Saale-Unstrut Organic; E3, Saale-Unstrut Conventional). Peak intensities were normalized to the peak intensity of the internal standard, deuterated phosphatidylethanolamine (d_7_ PE 15:0-18:1), and subsequently log-transformed. For each sample, values from five technical replicate injections were averaged before heatmap visualization (*n* = 5 technical replicate injections per sample). The color scale represents log-transformed, internal standard-normalized peak intensities, with red indicating high and blue indicating low relative abundance.

To evaluate the phytoceramide composition of the GlcCER-enriched precipitates obtained from the three apple pomace samples (E1, E2, and E3), each precipitate was analyzed by UHPLC-VIP-HESI-MS/MS. A total of 59 phytoceramides were putatively annotated in the GlcCER-enriched precipitates obtained from the three apple pomace sources, representing an 18% increase in the number of annotated phytoceramides compared with the raw extracts reported in Section 2.4. Removal of flavonoids, triterpenoids, steroids, and fatty acids through the fractionation step simplified the sample matrix, enabling improved ionization of molecules and thus improved and increased annotation of phytoceramides. This observation is in line with Williams et al., 2023 [35], who opined that strategies in sample preparation that clean up a matrix can reduce ion suppression and enhance analyte signals. A heatmap was subsequently generated to compare the relative abundances of the annotated phytoceramide species across the three samples (Figure 3). Hierarchical clustering of normalized sphingolipid relative abundances revealed distinct CER and GlcCER profiles among the three apple pomace samples (E1–E3). The heatmap showed clear differences in the relative abundance of both CER and GlcCER, indicating sample-specific variation in sphingolipid composition. The organic Bodensee sample (E1) exhibited a relatively balanced CER profile, with intermediate normalized relative abundances across most CER species. Several GlcCER, including GlcCer-d18:2 h22:0 (40), GlcCer-t18:1 h16:0 (45), GlcCer-t18:0 h24:0 (55) and GlcCer-t18:3 h24:0 (61) displayed comparatively higher intensities than in E2. Saale-Unstrut Organic (E2) was characterized by the highest relative abundances of numerous ceramides (Figure S3, Table S2, Supplementary Data). Higher relative abundance was observed for Cer-t18:0 h21:0 (20), Cer-t18:0 h22:0 (21), Cer-t18:0 h23:0 (22), Cer-t18:0 h24:0 (23), and Cer-t18:0 h25:0 (24), which formed the upper cluster of the heat map. Conversely, several GlcCERs exhibited relatively low relative abundances, particularly GlcCer-t18:1 h24:0 (4), GlcCer-d18:1 h22:0 (36), GlcCer-10-hydroxy-d18:2 h24:0 (53), GlcCer-7,10-dihydroxy-8-oxo-d18:0 h22:0 (58), and related GlcCER species. Saale-Unstrut Conventional (E3) samples displayed a phytoceramide profile distinct from both organic samples. Many of the CER species that were abundant in E2, including Cer-t18:0 h21:0 (20), Cer-t18:0 h22:0 (21), Cer-t18:0 h23:0 (22), Cer-t18:0 h24:0 (23), and Cer-t18:0 h25:0 (24), were present at relatively lower relative abundances in E3. In contrast, several GlcCERs, particularly GlcCer-t18:1 h23:0 (3), GlcCer-t18:1 h24:0 (4), GlcCer-t18:1 h16:0 (45), GlcCer-t18:1 h26:0 (50) and GlcCer-t18:0 h24:0 (55) exhibited higher relative abundances in E1 compared to E2 and E3 (Figure 3). Overall, the heatmap revealed distinct CER and GlcCER profiles among the three apple pomace samples, with differences observed between E1, E2, and E3 indicating variations in sphingolipid compositions. These patterns may be related to differences in geographical origin, cultivation system, and processing history, as well as other factors, such as apple cultivar, which were not investigated in this study.

### 2.8. Purification of the GlcCER-Enriched Precipitates

Because plant glucosylceramides (GlcCERs) have attracted increasing interest as bioactive cosmetic ingredients and natural precursors of ceramides, further purification of the GlcCER-enriched precipitates was undertaken to determine their molecular composition. This characterization is particularly important because different GlcCER molecular species generate structurally distinct ceramides following enzymatic or chemical hydrolysis, and these ceramide subclasses exhibit different physiological and cosmetic functions, including skin barrier restoration, hydration, elasticity, and epidermal homeostasis [36,37,38]. Consequently, detailed characterization of the major GlcCER subclasses is essential for assessing the quality and commercial value of apple pomace as a sustainable source of cosmetic sphingolipids. Based on this rationale, the GlcCER-enriched precipitates obtained from the extraction of E1, E2, and E3 were further purified by semi-preparative HPLC on a Merck HiBar C8 column, affording four pure GlcCERs (1–4) together with two semi-pure fractions (GlcCERF_0_ and GlcCERF_1_) (Figure S4, Supplementary Data). The recovery of the GlcCER-enriched precipitates during semi-preparative purification remained high for all three samples, reaching 76% (*w*/*w*) for E1, 72% (*w*/*w*) for E2, and 85% (*w*/*w*) for E3 (Table 1). These results demonstrate that the more sustainable purification workflow efficiently recovered the major GlcCER constituents while maintaining high purification yields.

A closer examination of the molecular composition revealed marked differences among the three samples. The sphingadienine-based GlcCER-AS 1 was the predominant molecular species in the organically grown E2 sample (99.7 mg), compared with the conventionally grown E3 sample (71.1 mg), despite identical extraction, fractionation, and purification procedures. This observation suggests that the variation primarily reflects intrinsic biological differences rather than methodological factors. Phytosphingoid-based GlcCERs, particularly the t18:1 species containing an (*E*)-4-hydroxy-8-sphingenine base, were represented mainly by compound 4 (83.9 mg), as well as compounds 2 and 3, making it the richest sample in this type of GlcCERs. These compositional differences are particularly relevant because ceramides with phytosphingoid backbones, including those derived from t18:1/(*E*)-4-hydroxy-8-sphingenine and t18:0 bases (e.g., NP and AP subclasses), are among the most widely used ceramide subclasses in cosmetic formulations owing to their recognized roles in restoring the epidermal lipid barrier, improving skin hydration and reducing transepidermal water loss [36,37,38]. More generally, the predominance of phytosphingoid-type GlcCERs in the studied apple pomaces enhances their value as a raw material for the production of cosmetic sphingolipids. The observed shifts in sphingoid base composition among E1, E2, and E3 are consistent with previous studies demonstrating that the molecular profile of plant GlcCERs may be influenced by geographical origin, genetic, environmental, agronomic, and processing-related factors [39,40,41].

### 2.9. UHPLC-VIP-HESI-MS/MS of the Fractions GlcCERF_0_ and GlcCERF_1_

The two semi-pure fractions, GlcCERF_0_ and GlcCERF_1_, obtained during the purification of the GlcCER-enriched precipitates were analyzed using an Agilent 1290 HPLC system coupled to a Bruker timsTOF Pro mass spectrometer equipped with a Vacuum Insulated Probe Heated Electrospray Ionization (VIP-HESI) source. Several GlcCER species were detected in both fractions (Table S2, Figure S3, Supplementary Data). In GlcCERF_0_, the main detected species included GlcCer-d18:2 h15:0 (38), GlcCer-d18:3 h16:0 (43), GlcCer-t18:1 h16:0 (45), GlcCer-7,10-dihydroxy-d18:2 h16:0 (56), GlcCer-7,10-dihydroxy-d18:2 h24:0 (57), and GlcCer-8-oxo-d18:2 h16:0 (62). GlcCERF1 contained a broader range of detected species, including GlcCer-d18:2 h18:0 (39), GlcCer-d18:2 h22:0 (40), GlcCer-d18:2 h24:0 (41), GlcCer-d18:3 h22:0 (44), GlcCer-t18:1 h20:0 (46), GlcCer-t18:1 h21:0 (47), GlcCer-t18:1 h25:0 (49), GlcCer-t18:1 h26:0 (50), GlcCer-10-hydroxy-d18:2 h24:0 (53), GlcCer-10-hydroxy-d18:2 h25:0 (54), GlcCer-t18:0 h24:0 (55), GlcCer-7,10-dihydroxy-8-oxo-d18:0 h22:0 (58), GlcCer-7,10-dihydroxy-8-oxo-d18:0 h24:0 (59), GlcCer-t18:3 h22:0 (60), and GlcCer-10-hydroxy-8-oxo-t18:0 h23:0 (63). Based on normalized peak intensities, GlcCer-t18:1 h16:0 (45) and GlcCer-t18:1 h21:0 (47) showed the highest relative abundance in GlcCERF_0_ and GlcCERF_1_, respectively. Together with the structural characterization of compounds 1–4, these results support the predominance of phytosphingoid-based GlcCERs among the glucosylceramides detected in apple pomace.

### 2.10. Total GlcCER Content of the Apple Pomace E1, E2 and E3

The total GlcCER content (TGC, mg/Kg of apple pomace) was calculated as the sum of the masses of all pure and semi-pure GlcCER fractions recovered after semi-preparative HPLC purification and normalized to 1.0 Kg of starting apple pomace. The TGC values were comparable for E1 (391 mg/Kg) and E3 (402 mg/Kg), whereas E2 contained a markedly lower amount (264 mg/Kg), indicating that total GlcCER accumulation is not determined solely by geographical origin but is also influenced by cultivation practices and/or industrial processing conditions. Reisberg et al. [27] reported a TGC of 289 mg/Kg of apple pomace using a conventional extraction protocol involving chlorinated solvents. In the present study, comparable (E2) and substantially higher GlcCER contents (E1 and E3) were obtained using a completely chlorinated solvent-free workflow.

The potential of apple pomace as a source of GlcCERs should also be considered in comparison with other established plant sources and agro-industrial residues. However, differences in extraction procedures, including solvent composition, extraction temperature, extraction time, and the number of extraction cycles, may influence GlcCER recovery and should therefore be considered when comparing reported yields across different raw materials and studies.

Takakuwa et al. [42] conducted a comparative survey of GlcCERs in a wide range of crop tissues and processing by-products using a chloroform–methanol extraction followed by treatment with methanolic KOH. Among the materials investigated, apple pulp showed the highest concentration with TGC of 940 mg/kg, followed by sunflower root (610 mg/kg), apple skin (490 mg/kg), sugar-beet pulp (450 mg/kg), reed canary grass (450 mg/kg), and grain amaranth stem (410 mg/kg). Other potentially relevant materials included soybean root (390 mg/kg), grain amaranth grain (390 mg/kg), adzuki bean cotyledon (340 mg/kg), sugar-beet stem (310 mg/kg), and sugar-beet fiber (290 mg/kg). In contrast, considerably lower concentrations were reported for potato tuber (10 mg/kg), potato pulp (20 mg/kg), buckwheat flour (10 mg/kg), soybean okara (10 mg/kg), and mushroom mat (60 mg/kg). These data illustrate the substantial variability in GlcCER abundance among plant species and, importantly, among different tissues or processing fractions of the same plant. Reisberg et al. [27] reported GlcCER contents of 337.0 and 44.0 mg/kg for wheat germ and coffee grounds, respectively. Recent studies also demonstrate that other agro-industrial residues may constitute interesting sources of plant sphingolipids. For example, Yaita et al. [43] recently quantified individual GlcCER and ceramide species in cocoa-processing materials by LC-ESI-MS/MS. The reported GlcCER species in cocoa bean shells corresponded to approximately 0.45 mg/g when summed, while cocoa germ contained approximately 0.77 mg/g.

The GlcCER content determined in the present study (260–400 mg/kg) is therefore within the range reported for these plant-derived residues, although direct comparison should be made cautiously because of differences in raw-material composition, processing history, extraction procedures, and analytical methodology.

### 2.11. Annotation Strategy of CERs and GlcCERs

Phytoceramides generate characteristic MS/MS fragmentation patterns that enable assignment of their sphingoid base (long-chain base, LCB) and fatty acid moieties based on diagnostic fragment ions associated with chain length, hydroxylation, degree of unsaturation, and glycosylation status (Figures S5–S8, Supplementary Data). These fragmentation features allow discrimination of GlcCER and CER species according to previously established lipidomic workflows [44,45,46]. GlcCER species showed neutral losses of *m*/*z* 162.051 (C_6_H_10_O_5_) and 180.061 (C_6_H_12_O_6_), corresponding to cleavage of the hexose moiety. Successive loss of water after the sugar loss enabled the determination of the number of hydroxyl groups on the sphingosine chain. By considering the sphingosine and fatty acid fragments alongside the mother ion, we were able to deduce the molecular formula of each GlcCER species, including the number of hydroxyl groups, degree of unsaturation, and, in rare cases, presence of a keto/oxo group (Figure 2 and Figure 3). Similarly, CER showed characteristic fragmentation patterns but without the hexose moiety (Figures S5–S8, Supplementary Data). Ion mobility mass spectrometry provided complementary structural confirmation through collision cross section (CCS) measurements. CCS values are influenced by molecular size, fatty acyl chain length, unsaturation, hydroxylation, and headgroup composition [47,48,49]. CER species displayed CCS values of approximately 250–290 Å^2^ (Table S2), whereas GlcCER species showed larger values of approximately 280–320 Å^2^, reflecting the increased molecular size and altered gas-phase conformation associated with glycosylation [50,51]. Increasing fatty acyl chain length resulted in higher CCS values, while hydroxylation produced smaller but measurable increases. Most experimental CCS values agreed within ±1–2% of reference or predicted values, supporting confident structural annotation (Table S2).

Based on the type of annotation, the predominant sphingoid bases among the phytoceramides were phytosphingenine (t18:1; 27%), sphingadienine (d18:2; 19%), and phytosphingosine (t18:0; 13%), consistent with major phytoceramide classes reported in plants [46]. The identified sphingoid bases included d18 and t18 species differing in hydroxylation and unsaturation (Figure S3, Supplementary Data), while fatty acid moieties were predominantly α-hydroxylated saturated chains ranging from C14 to C26, consistent with plant sphingolipid composition [39,52]. Only two unsaturated fatty acids were detected, corresponding to Cer t18:1 h26:1 (34) and GlcCer t18:1 h22:1 (48). Accurate mass measurements supported structural assignments, with most mass errors below 5 ppm (Table S2). Additional oxygenated phytoceramide derivatives containing 10–12 oxygen atoms were identified, including 8-keto GlcCER species and hydroxylated long-chain base-containing phytoceramides, further demonstrating the structural diversity of phytoceramides extracted from apple pomace (Figure S3, Supplementary Data). Overall, integration of UHPLC-VIP-HESI-MS/MS fragmentation with ion mobility measurements enabled robust characterization and discrimination of structurally related apple phytoceramide species, further revealing the complexity of apple pomace as a source of phytoceramides.

### 2.12. NMR Characterization of the Major GlcCERs (1–4)

To unequivocally confirm the identities of the major GlcCER molecular species putatively annotated by UHPLC-VIP-HESI-MS/MS, the structures of compounds 1–4 (Figure S3, Supplementary Data) were elucidated by comprehensive HRESIMS and 1D- and 2D-NMR spectroscopic analyses (Figures S9–S32, Tables S2 and S3, Supplementary Data) in comparison with published literature data.

Compounds 1–4 were isolated as a white powder soluble in pyridine. Their molecular formula were deduced from the analysis of their HRESIMS spectra displaying the protonated ion peaks: C_40_H_75_NO_9_ (1): [M+H]^+^ at *m*/*z* 714.5534 (calc. 714.5515 for C_40_H_76_NO_9_^+^); C_46_H_89_NO_10_ (2): [M+H]^+^ at *m*/*z* 816.6597 (calc. 816.6559 for C_46_H_90_NO_10_^+^); C_47_H_91_NO_10_ (3): [M+H]^+^ at *m*/*z* 830.6719 (calc. 830.6716 for C_47_H_92_NO_10_^+^) and C_48_H_93_NO_10_ (4): [M+H]^+^ at *m*/*z* 844.6886 (calc. 844.6872 for C_48_H_94_NO_10_^+^) respectively indicating the presence of GlcCER derivatives. Moreover, compounds 2–4 exhibited a successive increase of one carbon unit, suggesting homologous variations in fatty acid chain length.

Compounds 1–4 exhibited in ^1^H-NMR spectra a diagnostic secondary amide proton appearing as a doublet at δ_H_ = 8.38–8.61 (*J* ≈ 8.5–9.2 Hz), characteristic of a CER core structure (Figure S3, Table S3, Supplementary Data), with the corresponding carbonyl resonating between δ_C_ = 175–176 (Table 2). Importantly, both GlcCers with sphingoid (compound 1) and phytosphingoid (compounds 2–4) backbones shared a nitrogen-bearing methine at C-2 resonating between δ_C_ = 52–55, confirming the attachment to the amide nitrogen, while oxygenated carbons at C-1, C-3, and C-4 resonated between δ_C_ = 70–76, as confirmed by COSY, HSQC, and HMBC spectra. The 4-hydroxy-*trans*-8-sphingenine backbone (2–4) was clearly identified by three oxygenated carbons (C-1, C-3, C-4), whereas compound 1 lacks the C-4 hydroxyl and instead shows an olefinic carbon at δ_C_ = 132.5 (Table 2). The coupling constants further supported the stereochemical assignment: H-2/H-3 (*J* ≈ 5–6 Hz) and the characteristic multiplicities are consistent with the *erythro* configuration corresponding to (2*S*,3*R*) stereochemistry in 1, while the additional H-3/H-4 relationships in the GlcCERs 2–4 supported a (*2S*,*3S*,*4R*) configuration, in agreement with reported data for natural phytoceramides [38,53,54]. Olefinic protons at δ_H_ = 5.50–5.98 and carbons at δ_C_ = 129–132 indicated the presence of double bonds. Their *trans*-configurations were all confirmed by allylic carbon shifts between δ_C_ = 33–34, whereas *cis*-configured double bonds typically would show allylic carbons below δ_C_ = 30, providing a reliable criterion for stereochemical assignment [55]. The sphingoid base chain length in 1–4 was assigned as C-18 based on well-established biosynthetic pathways involving palmitoyl-CoA and serine, which consistently yield C-18 long-chain bases in plants [56,57]. In contrast, fatty acid chain lengths in 1–4 were determined by mass spectrometry. The stereochemistry of the 2-hydroxy fatty acids in 1–4 was assigned as (2′*R*), consistent with enzymatic stereoselectivity in natural phytoceramides and supported by the characteristic α-proton at δ_H_ = 4.60 (dd, *J* ≈ 8.0, 3.7 Hz) and δ_C_ = 72–73 [58]. Finally, compounds 1–4 share a glucopyranosyl unit, evidenced by an anomeric proton at δ_H_ = 4.94–4.97 (d, *J* ≈ 7.7–7.8 Hz) and δ_C_ = 106, indicative of *β*-configuration. The full set of sugar carbons (δ_C_ = 63–79), particularly C-4 (δ_C_ = 72–73 ppm), was consistent with glucose, in agreement with reported ^1^H and ^13^C-NMR assignments of monosaccharides [59,60]. Finally, the structures of the GlcCERs 1–4 were confirmed by the MS fragments observed on their HRESIMS spectra (Figures S5–S8, Supplementary Data). All these data in comparison with those published in the literature (Reisberg et al., 2017, Adem et al., 2021) [27,28] allowed to establish the structures of the most abundant GlcCERs (1–4) in apple pomace as: 1-*O*-β-_D_-glucopyranosyl-(*2S*,*3R*,*4E*,*8E*)-2-[(2’*R*)-2’-hydroxyhexadecanoylamino]octadeca-4,8-diene-1,3-diol or GlcCer-d18:2 h16:0^Δ4,8E^ (1), 1-*O*-β-_D_-glucopyranosyl-(*2S*,*3S*,*4R*,*8E*)-2-[(2’*R*)-2’-hydroxydocosanoylamino]octadec8-ene-1,3,4-triol or GlcCer-t18:1 h22:0^Δ8E^ (2), 1-*O*-*β*-_D_-glucopyranosyl-(*2S,3S,4R,8E*)-2-[(2’*R*)-2’-hydroxytricosanoylamino]octadec-8-ene-1,3,4-triol or GlcCer-t18:1 h23:0^Δ8E^ (3) and 1-*O*-*β*-_D_-glucopyranosyl-(*2S*,*3S*,*4R*,*8E*)-2-[(2’*R*)-2’-hydroxytetracosanoylamino]octadec-8-ene-1,3,4-triol or GlcCer-t18:1 h24:0^Δ8E^ (4) (Figure S3, Supplementary Data).

The structures of compounds (1–4) suggest that under reversed-phase HPLC conditions, the retention behavior of GlcCERs reflects the balance between the hydrophilic contributions of their sugar moiety and hydroxyl functionalities and the hydrophobic contribution of the ceramide backbone. In general, GlcCER species bearing longer fatty acyl chains may exhibit stronger hydrophobic interactions with the stationary phase and consequently longer retention times. However, retention also depends on the overall molecular structure, including the degree of unsaturation and the number and position of the functional groups.

### 2.13. Ecological and Socio-Economic Relevance of the Study

Beyond the analytical and methodological advances, this study highlights the potential of apple pomace valorization within a circular bioeconomy framework. By recovering GlcCERs from an abundant agro-industrial by-product, the proposed workflow may contribute to the higher-value utilization of this biomass as a complementary valorization route alongside established applications [61,62]. Furthermore, the use of non-chlorinated solvents, together with the elimination of chlorinated solvents and n-hexane traditionally employed in sphingolipid isolation, may reduce hazardous chemical emissions and waste while improving operator safety, in accordance with the principles of Green Chemistry [17,19,29]. However, the overall environmental and economic benefits of the process cannot be established without a comprehensive assessment of solvent demand, energy consumption, waste generation, recovery efficiency, and operating costs. The implementation of a more sustainable solvent-based workflow further reinforces this sustainability by eliminating chlorinated solvents traditionally used in lipid extraction. On a societal level, such valorization strategies could support new value chains from low-cost by-products, thereby promoting sustainable practices and local employment, and contributing to the United Nations Sustainable Development Goals (SDGs), particularly SDG 12 (Responsible Consumption and Production), SDG 9 (Industry, Innovation and Infrastructure), and SDG 3 (Good Health and Well-being) through the production of safer plant-derived ceramides for cosmetic applications using safer manufacturing processes [63]. Economically, the results demonstrate that apple pomace constitutes a non-negligible and competitive source of GlcCERs, particularly phytosphingoid-type GlcCERs, whose hydrolysis yields CERs widely used in the cosmetic industry. This is of particular importance given the increasing demand for plant-derived CERs as safer and more sustainable alternatives to animal-derived sources, especially bovine materials, which are associated with sanitary and ethical concerns [5]. The comparable or higher GlcCER yields obtained in this study using a greener process further underline the industrial potential of apple pomace as a renewable and eco-friendly feedstock to produce high-value cosmetic ingredients, although its competitiveness and industrial potential remain to be established.

## 3. Materials and Methods

### 3.1. General

The extractions of the apple pomaces were performed in a Bandelin Sonorex sonicator bath (electronic GmbH & Co. KG, Berlin, Germany) (Ultrasonic frequency: 35 kHz). The bath was set at 50 °C (methanol treatments) and 70 °C (isopropanol or ethanol treatments). The extraction time was set to 30 min for small extractions and 3 × 15 min for large-scale extractions. Filtrations were done using glass microfiber filters/VWR (VWR International GmbH, Darmstadt, Germany) (small-scale) and a vacuum filtration setup for large-scale. A rotary evaporator (Heidolph Instruments, Schwabach, Germany)was used for the concentration of the extract. The pump was maintained above 70 mmHg to avoid foaming. The extracts were completely dried using a freeze-drying process. Vacuum Liquid Chromatography (VLC) was performed using silica gel (0.015–0.040 mm) on a Buchner funnel with a filter frit (ϕ = 20 cm). Column chromatography was performed on Sephadex LH 20 (Fluka, Seelze, Germany). Thin-layer chromatography (TLC) analyses were performed on precoated silica gel F_254_ aluminum sheets eluted with ethyl acetate-isopropanol 85:15 (*v*/*v*) + 0.1% acetic acid. Analytes were visualized after spraying the plates with copper (II) sulfate (10%), phosphoric acid (8%), and methanol (5%) in water and heated at 150 °C for 10 min, or with vanillin (0.5 g), methanol (85 mL), acetic acid (10 mL), and conc. H_2_SO_4_ (5 mL). The semi-preparative HPLC was performed on a Shimadzu Prominence system (Kyoto, Japan), which consists of an SPD-M20A diode array detector, an FRC-10A fraction collector, a CBM-20A communications bus module, a DGU-20A5R degassing unit, an LC-20AT liquid chromatograph, and a SIL-20A HT autosampler. High-resolution ESI-MS (HRESIMS) spectra of the isolated compounds were acquired by direct injection through an autosampler (1 μL injection volume) using a SCIEX TripleTOF 6600 QTOF mass spectrometer (SCIEX, Concord, ON, Canada) equipped with an ESI DuoSpray ion source. Data were acquired in positive-ion mode over an *m*/*z* range of 100–2500 at a resolving power of approximately 30,000 (FWHM at *m*/*z* 400), with external calibration. Data acquisition and processing were performed using Analyst TF 1.7.1 and PeakView 2.2 (SCIEX), respectively. ^1^H and ^13^C NMR spectra were recorded on an Agilent DD2 400 NMR spectrometer (Santa Clara, CA, USA) at 399.917 and 100.570 MHz, respectively. ^1^H chemical shifts are referenced to internal TMS (^1^H δ = 0 ppm) and ^13^C chemical shifts to C_5_D_5_N (^13^C δ = 123.5 ppm). The HSQC experiment was optimized for ^1^*J*_CH_ = 146 Hz with ^13^C-decoupling during acquisition. The HMBC experiment was optimized for a long-range coupling of 8 Hz. Coupling constants (*J*) are reported in Hertz (Hz). Signal multiplicities are described as singlet (s), doublet (d), triplet (t), quartet (q), multiplet (m), or broad (br). Peak assignments were made based on chemical shift, integration, and multiplicity, and were supported by comparison with literature data. GlcCer-d18:2 h16:0 and Cer-d18:1 h18:0 from soybeans, purchased from (Avanti Polar Lipids, Inc., Alabaster, AL, USA), together with β-sitosterol glucoside obtained from the in-house physical compound library, were used as reference compounds.

### 3.2. Plant Material

Three dried apple pomace (*Malus domestica*) residues provided by Döhler Saale-Unstrut, obtained from two German apple tree growing regions (Bodensee and Saale-Unstrut), were used in this study. They underwent different apple juice processing methods, from the Bodensee region (E1), where organically grown apples were submitted to only one extraction/pressing step, and the apple pomace was available for analysis. From Saale-Unstrut, apple pomace residue was obtained by first extraction/pressing, followed by an enzymatic digestion and lastly a second extraction/pressing of organically grown apples (E2) and conventional farming (E3).

### 3.3. Physical Properties-Moisture Content

The moisture content (MC) was determined using the gravimetric (oven-drying) method, following standard protocols such as those described by AOAC International [64]. An empty, clean, pre-dried moisture dish was weighed (W_0_) using an analytical balance. The dish containing approximately 100 g of each sample was accurately weighed (W_1_). The samples were then dried in a hot air oven maintained at 105 ± 2 °C for 24 h until constant weight was achieved (W_2_). The experiment was carried out in three biological replicates. Moisture content (%) was calculated using the following equation:


$$\mathrm{MC}=\frac{\left(W1-W2\right)}{\left(W1-W0\right)}\times 100$$

### 3.4. Extraction, Fractionation and Purification

#### 3.4.1. Small-Scale Extractions

Ultrasound-assisted extraction (UAE) was performed on 1.0 g of each apple pomace sample (E1, E2 and E3) using ten solvent systems (AH–JH) at a fixed solid-to-solvent ratio of 1:6 (*w*/*v*). The ethanol- and isopropanol-based extractions (AH–GH) were carried out at 70 °C for 30 min, whereas the methanol-based extractions (HH–JH) were performed at 50 °C for 30 min. The solvent systems were as follows: AH, isopropanol–*n*-hexane–water (55:20:25, *v*/*v*/*v*) used by Reisberg et al., 2017 and Adem et al., 2021 as reference [27,28]; BH, ethanol–water (95:5, *v*/*v*); CH, ethanol–water (65:35, *v*/*v*); DH, ethanol–water (80:20, *v*/*v*); EH, isopropanol–water (95:5, *v*/*v*); FH, isopropanol–water (80:20, *v*/*v*); GH, isopropanol–water (65:35, *v*/*v*); HH, methanol–water (95:5, *v/v*); IH, methanol–water (80:20, *v*/*v*); and JH, methanol–water (65:35, *v*/*v*). After extraction, the marc was removed by filtration, and the filtrate was concentrated under reduced pressure at 50 °C. The resulting crude extract was subsequently freeze-dried and weighed. All experiments were performed in triplicate, and the extraction yields presented in Figure 1 are expressed as the mean ± standard deviation (SD).

#### 3.4.2. Isolation of the GlcCErs

##### Large-Scale Extractions

In accordance with the ICH Q3C guideline on residual solvents [16] and the principles of green chemistry, various protocols were evaluated to eliminate the use of Class 2 solvents (*n*-hexane, dichloromethane, and chloroform) during the production of GlcCER-enriched fractions. Therefore, 1.0 Kg of each apple pomace was extracted by sonication with 6 L of an ethanol–water mixture at 70 °C for 15 min × 3, corresponding to a solid-to-solvent ratio of 1:6 (*m*/*v*). The moisture content of each pomace was taken into account when adjusting the aqueous proportion of the extraction solvent. Accordingly, for E1, E2, and E3, containing 6.83 ± 0.11%, 6.21 ± 0.04%, and 4.00 ± 0.09% moisture, respectively, 4.80 L of ethanol and 1.132, 1.138, and 1.160 L of water, respectively, were added to 1 kg of pomace to provide an effective total extraction liquid volume of approximately 6 L with an 80:20 (*v*/*v*) ethanol–water composition. The marc was filtered and evaporated under reduced pressure to afford the crude extracts (Table 1 and Scheme S1, Supplementary Data).

##### Fractionation of the Crude Extracts

The crude extracts from E1, E2, and E3 were adsorbed onto 200 g of silica gel (0.015–0.04 mm) and dried overnight in an oven at 40 °C. The dried silica-adsorbed extracts were subsequently fractionated by vacuum liquid chromatography (VLC) using a Büchner funnel equipped with a 20 cm diameter filter frit. A slurry was prepared by suspending 500 g of silica gel (0.015–0.04 mm) in 2 L of ethyl acetate and subsequently poured into the Büchner funnel under vacuum. After packing, 1.25 L of solvent was recovered, resulting in a free silica bed volume of 0.75 L. The adsorbed extract was then loaded onto the silica bed. The 1.25 L of recovered ethyl acetate was adjusted to a final volume of 2.15 L and used to initiate the elution process. Elution was performed using only two solvent systems, yielding two subfractions: F_A_ (ethyl acetate–methanol 100:0, *v*/*v*; 2 L × 4) and F_B_ (ethyl acetate–methanol 92.5:7.5, *v*/*v*; 2 L × 4). To minimize solvent consumption, the solvents were recovered after each elution cycle and reused in the subsequent cycle, as described in Table S1 Supplementary Data.

##### Recovery of the GlcCER-Enriched Precipitates

Then, 360 g of dry Sephadex LH-20 was suspended in approximately 2 L of methanol and allowed to swell completely overnight. The swollen Sephadex was then gently homogenized and transferred into a 6 cm diameter column. The column was packed under a low and constant flow of methanol until a bed height of approximately 76 cm was obtained. Excess methanol collected during the swelling and packing steps was recovered and reused when appropriate. The packed column was maintained fully covered with methanol throughout the procedure to prevent drying of the Sephadex.

The packed Sephadex LH-20 column was equilibrated with methanol. Fraction FB was loaded in the minimum practical volume of methanol. Elution was performed with methanol at a flow rate of 2.5 mL/min. An average of 70 fractions of 14 mL each (approximately 980 mL of elution volume) were collected and pooled based on their TLC profiles (Figure S2, Supplementary Data) to afford the enriched GlcCER fraction (980.6 mg). The same protocol applied to pomaces E1 and E2 afforded 995.7 mg and 533.3 mg of GlcCER-enriched fractions, respectively.

The dried GlcCER-enriched fractions were treated twice with cold acetone–methanol (9:1, *v*/*v*; 150 mL × 2) at 4 °C for 8 h, followed by filtration, to give 473.8 mg, 516.0 mg, and 368.0 mg of GlcCER precipitates from E3, E1, and E2, respectively (Table 1), as beige powders.

##### Purification of the GlcCER Precipitate by HPLC

The enriched GlcCER precipitates from E1, E2, and E3 were purified, targeting the most abundant GlcCERs by semi-preparative HPLC using a Merck Hibar Säule C8 column (10 μm, 250 × 10 mm, Merck, Darmstadt, Germany) packed with LiChrospher material as the stationary phase. Solvent A consisted of deionized water (Milli-Q, Millipore, Schwalbach, Germany) containing 0.1% formic acid (FA), and solvent B consisted of HPLC-grade methanol containing 0.1% formic acid. The flow rate was set at 2.5 mL/min, and UV detection was performed at λ = 210 and 215 nm using the following elution gradient: isocratic at 95% solvent B for 10 min, increased to 97% at 14 min, held isocratic at 97% for 9 min, then decreased to 95% over 1 min, followed by equilibration at 95% for 4 min. GlcCERs 1 (*t*_R_ = 14.2 min), 2 (*t*_R_ = 20.9 min), 3 (*t*_R_ = 22.5 min), and 4 (*t*_R_ = 24.9 min) were isolated as white powders together with the enriched fractions GlcCERF_0_ (*t*_R_ = 8.8–13.4 min) and GlcCERF_1_ (*t*_R_ = 14.8–20.0 min).

### 3.5. CER and GlcCER Analysis by LC-timsTOF-MS

For the LC-MS investigations, 3.0 mg of the crude extract obtained in Section 3.4.1 was dissolved in 1.5 mL of methanol spiked with 100 nM of d_7_ PE (deuterated phosphatidylethanolamine, 15:0–18:1) as an internal standard (Avanti Research) in an ultrasonic bath. After centrifugation at 5000 rpm for 10 min, the solution was filtered with Syringe Filter H-PTFE/Altmann Analytik (13 or 25 mm) and submitted to LC-MS analysis at a final concentration of 2 mg/mL. Samples were analyzed using an Agilent Technologies 1290 Liquid Chromatography system (Agilent, Waldbronn, Germany) coupled to a Bruker timsTOF pro mass analyzer with a vacuum-insulated probe heated electron spray ionizer (Bruker, Bremen, Germany). 5 µL of each sample extract was injected onto a C_18_ column (Bruker Intensity Solo C_18_, 100 × 2.1 mm, 1.8 µm) at 55 °C. The separation was achieved through a gradient elution employing acetonitrile:water (60:40) (phase A) and isopropanol:acetonitrile (90:10) (phase B), both buffered with 0.1% (*v*/*v*) formic acid and 10 mM ammonium formate, at a constant flow rate of 0.45 mL/min. The elution gradient started with 40% B and increased for 8 min to 99% B, which was then maintained for 3.5 min before decreasing back to 40% B for 3 min. Mass spectrometric detection was performed in positive VIP-HESI mode employing a full scan acquisition in a range from 100 to 1350 *m*/*z*. The ion mobility acquisition window covered ion inverse reduced mobility values between 0.70 and 1.80 V·s/cm^2^ with a ramp time of 100 milliseconds (10 mobility scans/sec) and the MS averaging set to 1, leading to a high temporal resolution. The accumulation time and duty cycle were also set to 100 milliseconds each to ensure continuous parallel accumulation and analysis. The electric field gradient across the mobility cells (ramp rate) was set to 9.42 Hz, indicating that a total of 9.42 data points were acquired per cycle. UHPLC-VIP-HESI-MS/MS data processing and metabolite annotation were performed using MetaboScape version 2025b [65]. Annotation of lipids was done using the Lipid annotation tool provided by the Metaboscape software [66], and annotations were curated manually. For compound visualization, the feature list was exported in Excel format, and peak intensities were plotted as a heatmap or bar graph.

## 4. Conclusions

In this study, an integrated and chlorinated-solvent-free workflow was successfully developed for the extraction, fractionation, and purification of ceramides (CERs) and glucosylceramides (GlcCERs) from apple pomace using a comparatively more sustainable process. By avoiding chlorinated solvents and *n*-hexane traditionally employed in sphingolipid isolation, the proposed workflow represents a step toward safer and more environmentally sustainable processing in accordance with Green Chemistry principles. The proposed three-step process combines high extraction efficiency with sustainable downstream purification, providing GlcCER recoveries comparable to or higher than those achieved by conventional protocols relying on hazardous and chlorinated solvents. Structural and quantitative analyses demonstrated that apple pomace is particularly rich in phytosphingoid-type GlcCERs, while revealing differences in sphingolipid composition among samples that may be associated with geographical origin, cultivation practices, and apple processing history. Other factors, such as apple cultivar, which were not investigated in this study, may also contribute to the observed variation in CER and GlcCER composition and, consequently, the potential cosmetic value of the recovered fractions. The combination of waste valorization, environmentally benign solvent systems, and efficient purification illustrates how Green Chemistry and Circular Bioeconomy principles can be successfully integrated into the production of high-value natural products from renewable agro-industrial residues. The developed workflow provides a practical platform for the valorization of apple pomace, which can be extended to other agricultural by-products, thereby supporting the transition toward safer, circular, and bio-based manufacturing of high-value natural ingredients while contributing to the United Nations Sustainable Development Goals related to responsible consumption and production (SDG 12), sustainable industrial innovation (SDG 9), and good health and well-being (SDG 3) through the sustainable process for the production of safer plant-derived cosmetic ingredients. However, further investigations addressing process reproducibility at larger scales, energy and solvent requirements, solvent recovery efficiency, waste generation, operating costs, and process optimization will be required to assess its potential for industrial implementation.

## Figures and Tables

**Table 1:** GlcCER concentrations (mg/Kg of apple pomace) in the three apple pomace samples (E1, E2 and E3).

Compound/Apple Pomace	E1 (Bodensee)	E2 (Saale-Unstrut)	E3 (Saale-Unstrut)
Extract mass (g)	225	140	164
**Fractions**			
GlcCER-enriched fraction (mg)	996	533	981
GlcCER precipitate (mg)	516	368	474
**Semi-pure GlcCERs**			
GlcCERF_0_ (mg)	49.0	24.3	39.8
GlcCERF_1_ (mg)	76.4	28.0	94.8
**Pure GlcCERs**			
GlcCER 1 (mg)	81.0	99.7	71.1
GlcCER 2 (mg)	70.7	39.4	74.9
GlcCER 3 (mg)	54.2	19.1	37.9
GlcCER 4 (mg)	59.3	53.4	83.9
**Total GlcCERs (mg/1000 g of apple pomace)**	**390.6**	**263.9**	**402.4**

**Table 2:** ^13^C-NMR data (100 MHz, Pyridine-*d*_5_, ẟ in ppm) of compounds 1–4.

Pos.	1	2	3	4
	ẟ_C_ (m)	ẟ_C_ (m)	ẟ_C_ (m)	ẟ_C_ (m)
Sphingoid base				
1	70.6 (t)	70.9 (t)	70.9 (t)	70.9 (t)
2	55.1 (d)	52.2 (d)	52.2 (d)	52.2 (d)
3	72.0 (d)	76.4 (d)	76.4 (d)	76.4 (d)
4	132.5 (d)	72.9 (d)	72.9 (d)	72.9 (d)
5	132.6 (d)	34.5 (t)	34.4 (t)	34.4 (t)
6	33.2 (t)	28.0 (t)	28.1 (t)	28.0 (t)
7	33.4 (t)	33.8 (t)	33.7 (t)	33.8 (t)
8	129.9 (d)	130.7 (d)	130.7 (d)	130.7 (d)
9	131.1 (d)	130.9 (d)	130.9 (d)	130.9 (d)
10	33.4 (t)	33.5 (t)	33.5 (t)	33.4 (t)
11	30.5 (t)	30.0–30.5 (t)	30.1–30.6 (t)	30.0–30.5 (t)
12–15	30.1–30.5 (t)	30.0–30.5 (t)	30.1–30.6 (t)	30.0–30.5 (t)
16	32.6 (t)	32.6 (t)	32.6 (t)	32.6 (t)
17	23.4 (t)	23.4 (t)	23.4 (t)	23.4 (t)
18	14.1 (q)	14.7 (q)	14.8 (q)	14.8 (q)
Fatty acid				
1′	176.2 (s)	176.1 (s)	176.1 (s)	176.1 (s)
2′	73.0 (d)	72.9 (d)	72.9 (d)	72.9 (d)
3′	36.1 (t)	36.0 (t)	36.0 (t)	36.0 (t)
4′	26.4 (t)	26.3 (t)	26.3 (t)	26.3 (t)
5′	28.1 (t)	28.4 (t)	28.4 (t)	28.3 (t)
6′–n′ ^a^	30.1–30.5 (t)	30.0–30.5 (t)	30.1–30.6 (t)	30.0–30.5 (t)
ω-2	32.6 (t)	32.6 (t)	32.6 (t)	32.6 (t)
ω-1	23.4 (t)	23.4 (t)	23.4 (t)	23.4 (t)
ω-CH3	14.1 (q)	14.7 (q)	14.7 (q)	14.8 (q)
Glucoside				
1″	106.1 (d)	106.0 (d)	106.1 (d)	106.1 (d)
2″	75.6 (d)	75.6 (d)	75.6 (d)	75.6 (d)
3″	78.9 (d)	78.9 (d)	78.9 (d)	78.9 (d)
4″	72.8 (d)	72.9 (d)	72.9 (d)	72.9 (d)
5″	79.0 (d)	79.0 (d)	79.0 (d)	79.0 (d)
6″	63.1 (t)	63.1 (t)	63.1 (t)	63.1 (t)

^a^ compound 1 (*n*′ = 13), compound 2 (*n*′ = 19), compound 3 (*n*′ = 20), compound 4 (*n*′ = 21).

## Data Availability

The data supporting the findings of this study are available from the corresponding author upon reasonable request.

## Supplementary Materials

The following supporting information can be downloaded at https://www.mdpi.com/article/10.3390/molecules31213807/s1, Figure S1: Moisture content of three apple pomace residues Bodensee Organic (E1), Saale-Unstrut Organic (E2), and Saale-Unstrut Conventional (E3). Bars represent mean ± SD, with individual data points shown (*n* = 3). Different lowercase letters indicate significant differences among apple pomace sources, as determined by one-way ANOVA followed by Tukey’s HSD test (*p* < 0.05); Figure S2: TLC profile (eluent: EtOAc–IPA, 85:15, *v*/*v* +0.1% acetic acid) of Sephadex LH-20 fractions showing enrichment of glucosylceramides with GlcCer-d18:2 h16:0 (Reference [1]) and β-sitosterol glucoside (Reference [2]); Figure S3: Putative structures of the annotated ceramide and glucosylceramide molecular species (1–64); Figure S4: Semi-preparative HPLC chromatogram of the GlcCer-enriched precipitate showing the isolation of compounds 1–4 and the enriched fractions GlcCerF_0_ and GlcCerF_1_; Figure S5: Suggested VIP-HESI/MS fragmentation pattern of GlcCer 1. Ion was extracted as extracted ions [M+H]^+^, compound numbers as given in Table S2; Figure S6: Suggested VIP-HESI/MS fragmentation pattern of GlcCer 2. Ion was extracted as extracted ions [M+H]^+^, compound numbers as given in Table S2; Figure S7: Suggested VIP-HESI/MS fragmentation pattern of GlcCer 3. Ion was extracted as extracted ions [M+H]^+^, compound numbers as given in Table S2; Figure S8: Suggested VIP-HESI/MS fragmentation pattern of GlcCer 4. Ion was extracted as extracted ions [M+H]^+^, compound numbers as given in Table S2; Figure S9: HRESIMS of compound 1; Figure S10: ^1^H-NMR spectrum (Pyridine-d_5_, 400 MHz) of compound 1; Figure S11: ^13^C-NMR spectrum (Pyridine-d_5_, 100 MHz) of compound 1; Figure S12: ^1^H-^1^H gDQCOSY spectrum of compound 1; Figure S13: ^1^H,^13^C gHSQCAD spectrum of compound 1; Figure S14: ^1^H,^13^C gHMBCAD spectrum of compound 1; Figure S15: HRESIMS of compound 2; Figure S16: ^1^H-NMR spectrum (Pyridine-d_5_, 400 MHz) of compound 2; Figure S17: ^13^C-NMR spectrum (Pyridine-d_5_, 100 MHz) of compound 2; Figure S18: ^1^H-^1^H gDQCOSY spectrum of compound 2; Figure S19: ^1^H,^13^C gHSQCAD spectrum of compound 2; Figure S20: ^1^H,^13^C gHMBCAD spectrum of compound 2; Figure S21: HRESIMS of compound 3; Figure S22: ^1^H-NMR spectrum (Pyridine-d_5_, 400 MHz) of compound 3; Figure S23: ^13^C-NMR spectrum (Pyridine-d_5_, 100 MHz) of compound 3; Figure S24: ^1^H-^1^H gDQCOSY spectrum of compound 3; Figure S25: ^1^H,^13^C gHSQCAD spectrum of compound 3; Figure S26: ^1^H,^13^C gHMBCAD spectrum of compound 3; Figure S27: HRESIMS of compound 4; Figure S28: ^1^H-NMR spectrum (Pyridine-d_5_, 400 MHz) of compound 4; Figure S29: ^13^C-NMR spectrum (Pyridine-d_5_, 100 MHz) of compound 4; Figure S30: ^1^H-^1^H gDQCOSY spectrum of compound 4; Figure S31: ^1^H,^13^C gHSQCAD spectrum of compound 4; Figure S32: ^1^H,^13^C gHMBCAD spectrum of compound 4; Scheme S1:Three-step fast, cost-effective, and green workflow for the production of a GlcCER enriched precipitates from the three apple pomace crude extracts; Table S1: Solvent use and recovery during vacuum liquid chromatography (VLC); Table S2: UHPLC-VIP-HESI-MS/MS characterization of ceramides and glucosylceramides in apple pomace; Table S3: ^1^H-NMR data (400 MHz, Pyridine-d_5_, ẟ in ppm) of compounds 1–4.

## Abbreviations

The following abbreviations are used in this manuscript:

BSE	Bovine spongiform encephalopathy
CER	Ceramide
d	Doublet
EAE	Enzyme-assisted extraction
GlcCER	Glucosylceramide
HESI	Heated electrospray ionization
HPLC	High-performance liquid chromatography
HRESIMS	High-resolution electrospray ionization mass spectrometry
m	Multiplet
MAE	Microwave-assisted extraction
MS	Mass spectrometry
MS/MS	Tandem mass spectrometry
NMR	Nuclear magnetic resonance
PLE	Pressurized liquid extraction
q	Quadruplet
s	Singlet
SFE	Supercritical fluid extraction
t	Triplet
UAE	Ultrasound-assisted extraction
UHPLC	Ultra-high-performance liquid chromatography
VIP	Vacuum Insulated Probe
VOC	Volatile organic compound
