# Supplementary material for: A Sustainable Workflow for the Recovery of Glucosylceramides from Apple Pomace
Source: Molecules. 2026 Oct 22;31(21):3807. doi: 10.3390/molecules31213807 (PMC13764739; doi:10.3390/molecules31213807)
Supplement: Supplementary file 1 [file molecules-31-03807-s001.zip › molecules-4596282-supplementary.pdf]

SUPPLEMENTARY DATA

A Sustainable Workflow for the Recovery of Glucosylceramides from Apple Pomace

Ghislain Fotso Wabo [1,*], Samuel Ngure Kariithi [2,*], Norbert Arnold [1], Jörg Ziegler [2] and Bernhard Westermann [1,3]

[1] Department of Bioorganic Chemistry, Leibniz Institute of Plant Biochemistry, Weinberg 3, D-06120 Halle (Saale), Germany; norbert.arnold@ipb-halle.de (N.A.); bernhard.westermann@ipb-halle.de (B.W.)
[2] Program Center for Plant Metabolomics and Computational Biochemistry, Leibniz Institute of Plant Biochemistry, Weinberg 3, D-06120 Halle (Saale), Germany; joerg.ziegler@ipb-halle.de
[3] Institute of Chemistry, Martin-Luther-University Halle-Wittenberg, Kurt-Mothes-Strasse 2, D-06120 Halle (Saale), Germany
* Correspondence: gfotsowa@ipb-halle.de (G.F.W.); samuelngure.kariithi@ipb-halle.de (S.N.K.)

Table of contents

Figure S1: Moisture content of three apple pomace residues Bodensee Organic (E1), Saale-Unstrut Organic (E2), and Saale-Unstrut Conventional (E3). Bars represent mean ± SD, with individual data points shown (*n* = 3). Different lowercase letters indicate significant differences among apple pomace sources, as determined by one-way ANOVA followed by Tukey's HSD test ($p < 0.05$). 4

Scheme S1:Three-step fast, cost-effective, and green workflow for the production of a GlcCER enriched precipitates from the three apple pomace crude extracts. 4

Table S1: Solvent use and recovery during vacuum liquid chromatography (VLC) 5

Figure S2: TLC profile (eluent: EtOAc–IPA, 85:15, v/v +0.1% acetic acid) of Sephadex LH-20 fractions showing enrichment of glucosylceramides with GlcCer-d18:2 h16:0 (Reference 1) and β-sitosterol glucoside (Reference 2). 5

Figure S3: Putative structures of the annotated ceramide and glucosylceramide molecular species (1–64). aOnly in extracts; bOnly in fractions; cExact position of the substituents and stereochemistry not known 8

Table S2: UHPLC-VIP-HESI-MS/MS characterization of ceramides and glucosylceramides in apple pomace. 8

Figure S4: Semi-preparative HPLC chromatogram of the GlcCer-enriched precipitate showing the isolation of compounds 1–4 and the enriched fractions $GlcCerF_0$ and $GlcCerF_1$. 10

Figure S5: Suggested VIP-HESI/MS fragmentation pattern of GlcCer 1. Ion was extracted as extracted ions $[M+H]^+$, compound numbers as given in Table S2. 10

Figure S6: Suggested VIP-HESI/MS fragmentation pattern of GlcCer 2. Ion was extracted as extracted ions $[M+H]^+$, compound numbers as given in Table S2. 11

Figure S7: Suggested VIP-HESI/MS fragmentation pattern of GlcCer 3. Ion was extracted as extracted ions $[M+H]^+$, compound numbers as given in Table S2. 11

Figure S8: Suggested VIP-HESI/MS fragmentation pattern of GlcCer 4. Ion was extracted as extracted ions $[M+H]^+$, compound numbers as given in Table S2. 11

Compound 1 12

Figure S9: HRESIMS of compound 1 12

Figure S10: ^{1}H-NMR spectrum (Pyridine-d_5, 400 MHz) of compound 1 12

Figure S11: ^{13}C-NMR spectrum (Pyridine-d_5, 100 MHz) of compound 1 13

Figure S12: ^{1}H-^{1}H gDQCOSY spectrum of compound 1 13

Figure S13: ^{1}H,^{13}C gHSQCAD spectrum of compound 1 14

Figure S14: ^{1}H,^{13}C gHMBCAD spectrum of compound 1 14

Compound 2 15

Figure S15: HRESIMS of compound 2 15

Figure S16: ^{1}H-NMR spectrum (Pyridine-d_5, 400 MHz) of compound 2 15

Figure S17: ^{13}C-NMR spectrum (Pyridine-d_5, 100 MHz) of compound 2 16

Figure S18: ^{1}H-^{1}H gDQCOSY spectrum of compound 2 16

Figure S19: ^{1}H,^{13}C gHSQCAD spectrum of compound 2 17

Figure S20: ^{1}H,^{13}C gHMBCAD spectrum of compound 2 17
Compound 3 18
Figure S21: HRESIMS of compound 3 18
Figure S22: ^{1}H-NMR spectrum (Pyridine-d$_5$, 400 MHz) of compound 3 18
Figure S23: ^{13}C-NMR spectrum (Pyridine-d$_5$, 100 MHz) of compound 3 19
Figure S24: ^{1}H-^{1}H gDQCOSY spectrum of compound 3 19
Figure S25: ^{1}H,^{13}C gHSQCAD spectrum of compound 3 20
Figure S26: ^{1}H,^{13}C gHMBCAD spectrum of compound 3 20
Compound 4 21
Figure S27: HRESIMS of compound 4 21
Figure S28: ^{1}H-NMR spectrum (Pyridine-d$_5$, 400 MHz) of compound 4 21
Figure S29: ^{13}C-NMR spectrum (Pyridine-d$_5$, 100 MHz) of compound 4 22
Figure S30: ^{1}H-^{1}H gDQCOSY spectrum of compound 4 22
Figure S31: ^{1}H,^{13}C gHSQCAD spectrum of compound 4 23
Figure S32: ^{1}H,^{13}C gHMBCAD spectrum of compound 4 23
Table S3: ^{1}H-NMR data (400 MHz, Pyridine-d_5, δ in ppm) of compounds 1-4 24

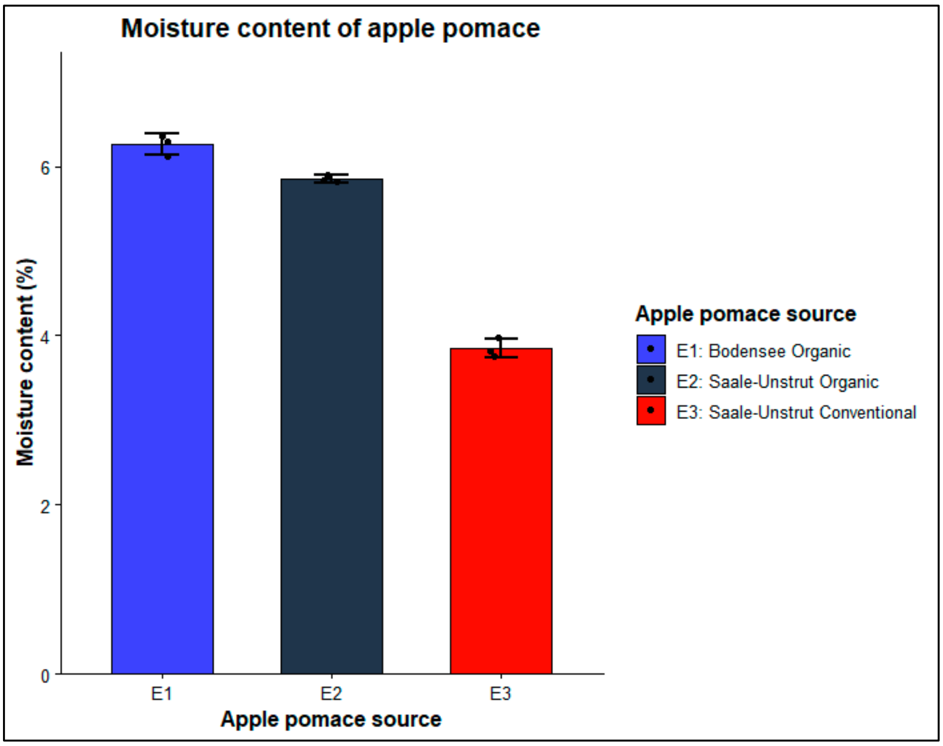

Figure S1: Moisture content of three apple pomace residues Bodensee Organic (E1), Saale-Unstrut Organic (E2), and Saale-Unstrut Conventional (E3). Bars represent mean ± SD, with individual data points shown (*n* = 3). Different lowercase letters indicate significant differences among apple pomace sources, as determined by one-way ANOVA followed by Tukey's HSD test ($p < 0.05$).

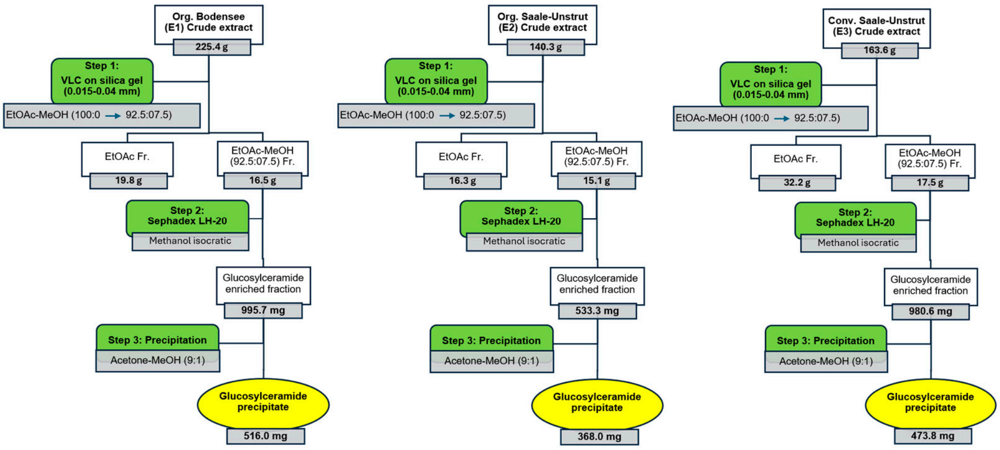

Scheme 1: Three-step fast, cost-effective, and green workflow for the production of a GlcCER enriched precipitates from the three apple pomace crude extracts.

Table S1: Solvent use and recovery during vacuum liquid chromatography (VLC)

Steps	Operation	EtOAc adsorbed/required/added	MeOH required/added	Fraction volume	Solvent recovered after evaporation (~ 95%)
F_A – Cycle 1	EtOAc 100%	2.150 L	–	2.000 L	1.900 L
F_A – Cycle 2	Recovered EtOAc + makeup to 2 L	0.100 L	–	2.000 L	1.900 L
F_A – Cycle 3	Recovered EtOAc + makeup to 2 L	0.100 L	–	2.000 L	1.900 L
F_A – Cycle 4	Recovered EtOAc + makeup to 2 L	0.100 L	–	2.000 L	1.900 L
Conversion $F_A \rightarrow F_B$	Adjust 1.900 L recovered EtOAc to 2 L of 92.5:7.5	–	0.1540 L	2.000 L	–
F_B – Cycle 1	EtOAc/MeOH 92.5:7.5, 2 L	–	0.0608 L	2.000 L	1.900 L
F_B – Cycle 2	Makeup to 2 L	0.0925 L	0.0075 L	2.000 L	1.900 L
F_B – Cycle 3	Makeup to 2 L	0.0925 L	0.0075 L	2.000 L	1.900 L
F_B – Cycle 4	Makeup to 2 L	0.0925 L	0.0075 L	2.000 L	1.900 L
**Total solvent used for the elution**		**~ 2.75 L**	**~ 0.25 L**		

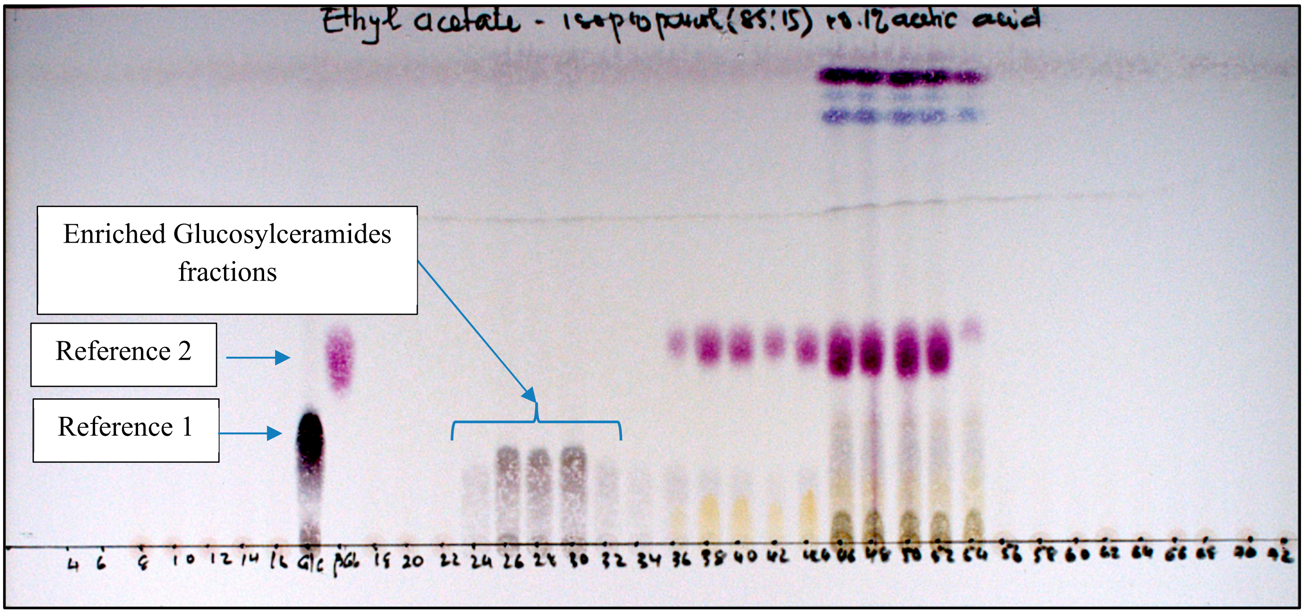

Figure S2: TLC profile (eluent: EtOAc–IPA, 85:15, v/v +0.1% acetic acid) of Sephadex LH-20 fractions showing enrichment of glucosylceramides with GlcCer-d18:2 h16:0 (Reference 1) and β-sitosterol glucoside (Reference 2).

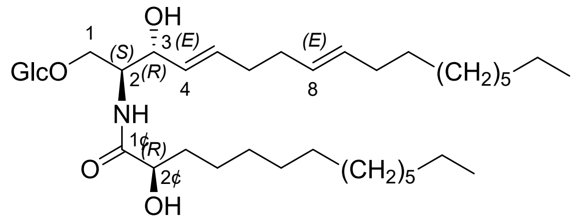

1. GlcCer-d18:2 h16:0 (n = 7)

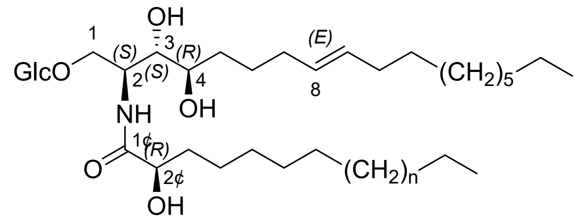

2. GlcCer-t18:1 h22:0 (n = 13)
3. GlcCer-t18:1 h23:0 (n = 14)
4. GlcCer-t18:1 h24:0 (n = 15)

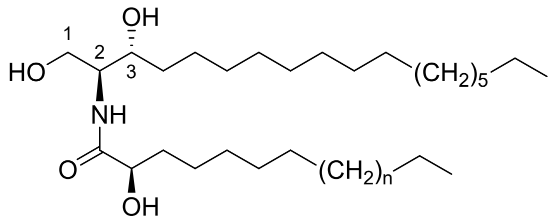

5. Cer-d18:0 h16:0 (n = 7)
6[b]. Cer-d18:0 h22:0 (n = 13)
7. Cer-d18:0 h26:0 (n = 17)

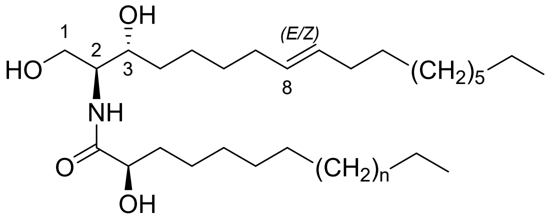

8[b]. Cer-d18:1 h16:0 (n = 7)
9[a]. Cer-d18:1 h22:0 (n = 13)
10. Cer-d18:1 h24:0 (n = 15)

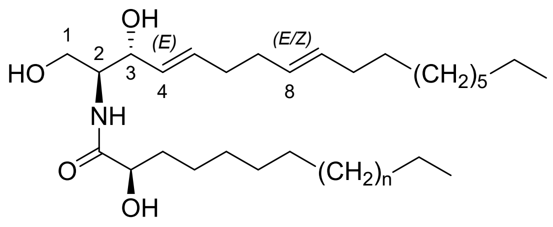

11. Cer-d18:2 h16:0 (n = 7)
12. Cer-d18:2 h22:0 (n = 13)
13. Cer-d18:2 h24:0 (n = 15)
14[a]. Cer-d18:2 h25:0 (n = 16)
15[a]. Cer-d18:2 h26:0 (n = 17)

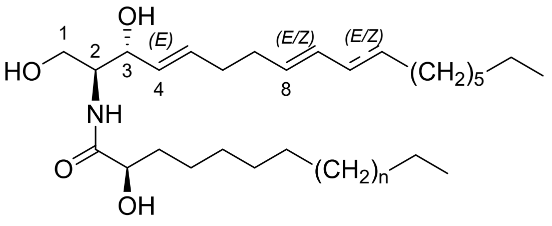

16[b]. Cer-d18:3 h16:0 (n = 7)

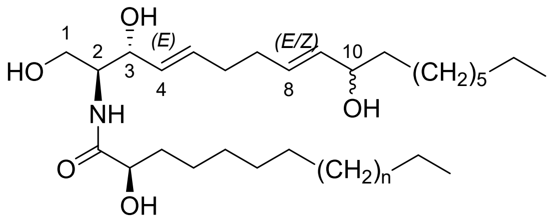

17[b c]. Cer-10-hydroxy-d18:2 h16:0 (n = 7, ω-9)
18[c]. Cer-10-hydroxy-d18:2 h24:0 (n = 15)

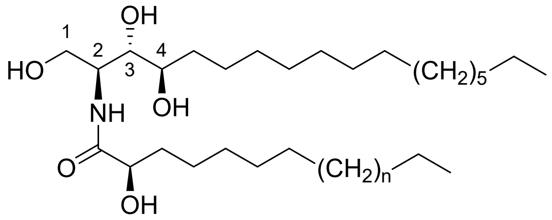

19. Cer-t18:0 h16:0 (n = 7)
20. Cer-t18:0 h21:0 (n = 12)
21. Cer-t18:0 h22:0 (n = 13)
22. Cer-t18:0 h23:0 (n = 14)
23. Cer-t18:0 h24:0 (n = 15)
24. Cer-t18:0 h25:0 (n = 16)
25. Cer-t18:0 h26:0 (n = 17)

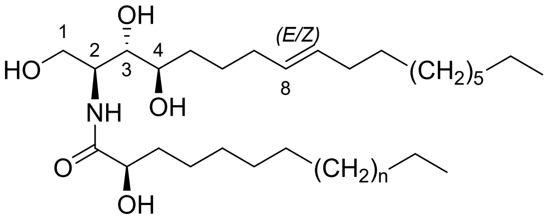

26. Cer-t18:1 h16:0 (n = 7)
27. Cer-t18:1 h20:0 (n = 11)
28[a]. Cer-t18:1 h21:0 (n = 12)
29. Cer-t18:1 h22:0 (n = 13)
30[a]. Cer-t18:1 h23:0 (n = 14)
31[b]. Cer-t18:1 h24:0 (n = 15)
32. Cer-t18:1 h25:0 (n = 16)
33. Cer-t18:1 h26:0 (n = 17)
34[a]. Cer-t18:1 h26:1 (n = 15, ω-9)

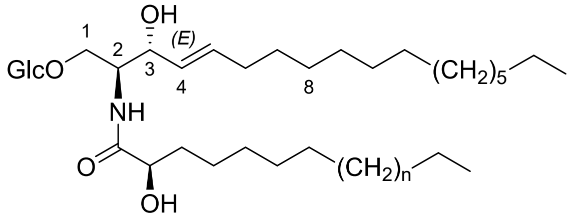

35. GlcCer-d18:1 h16:0 (n = 7)
36[b]. GlcCer-d18:1 h22:0 (n = 13)

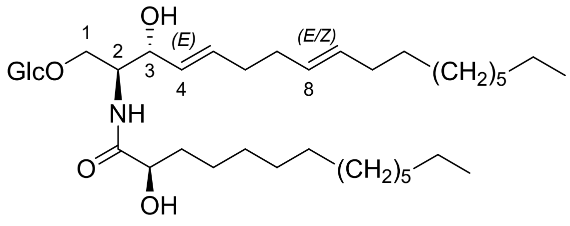

37[b]. GlcCer-d18:2 h14:0 (n = 5)
38[b]. GlcCer-d18:2 h15:0 (n = 6)
39. GlcCer-d18:2 h18:0 (n = 9)
40. GlcCer-d18:2 h22:0 (n = 13)
41. GlcCer-d18:2 h24:0 (n = 15)
42[b]. GlcCer-d18:2 h26:0 (n = 17)

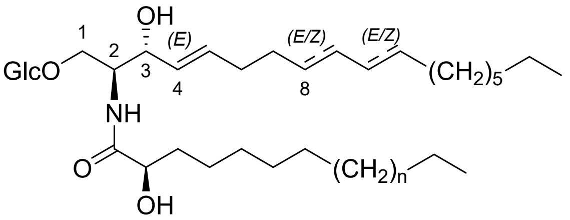

43. GlcCer-d18:3 h16:0 (n = 7)
44. GlcCer-d18:3 h22:0 (n = 13)

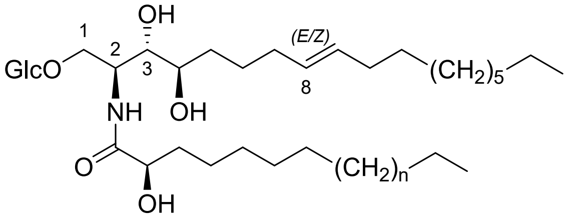

45. GlcCer-t18:1 h16:0 (n = 7)
46. GlcCer-t18:1 h20:0 (n = 11)
47. GlcCer-t18:1 h21:0 (n = 12)
48[b]. GlcCer-t18:1 h22:1 (n = 13, ω-9)
49. GlcCer-t18:1 h25:0 (n = 16)
50. GlcCer-t18:1 h26:0 (n = 17)

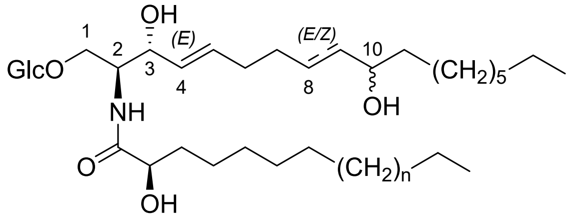

51[b c]. GlcCer-10-hydroxy-d18:2 h14:0 (n = 5)
52[b c]. GlcCer-10-hydroxy-d18:2 h16:0 (n = 7)
53. GlcCer-10-hydroxy-d18:2 h24:0 (n = 15)
54. GlcCer-10-hydroxy-d18:2 h25:0 (n = 16)

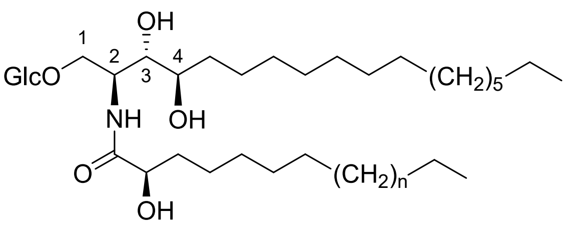

55. GlcCer-t18:0 h24:0 (n = 15) = 56

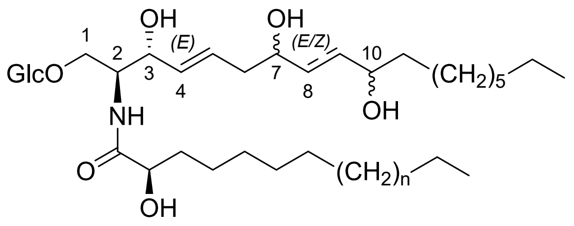

56[c]. GlcCer-7,10-dihydroxy-d18:2 h16:0 (n = 7)
57[c]. GlcCer-7,10-dihydroxy-d18:2 h24:0 (n = 15)

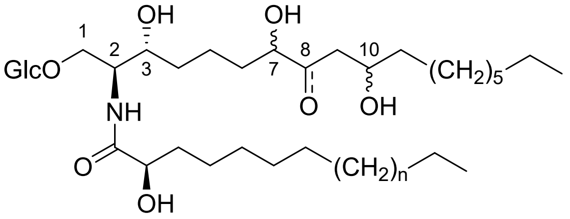

58[c]. GlcCer-7,10-dihydroxy-8-oxo-d18:0 h22:0 (n = 13)
59[c]. GlcCer-7,10-dihydroxy-8-oxo-d18:0 h24:0 (n = 15)

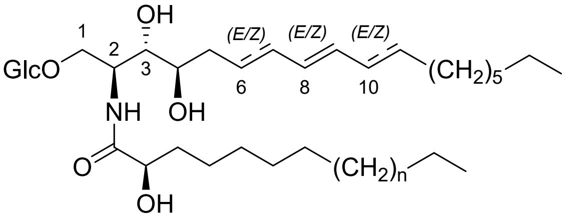

60[b]. GlcCer-t18:3 h22:0 (n = 13)
61[b]. GlcCer-t18:3 h24:0 (n = 15)

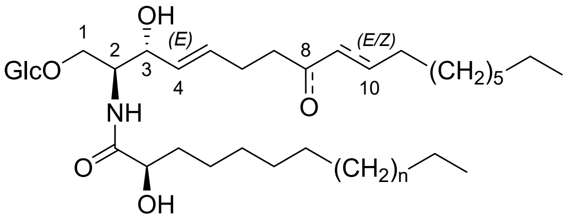

62[b c]. GlcCer-8-oxo-d18:2 h16:0 (n = 7)

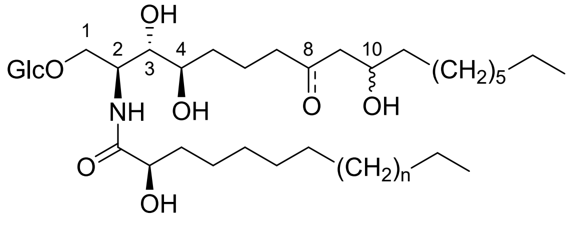

63[c]. GlcCer-10-hydroxy-8-oxo-t18:0 h23:0 (n = 14)

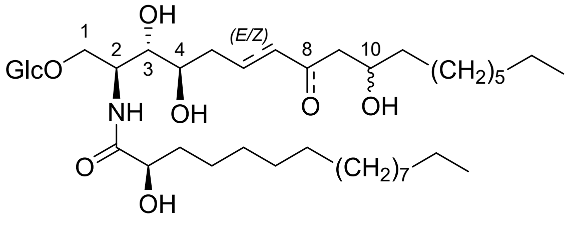

64[b c]. GlcCer-10-hydroxy-8-oxo-t18:1 h16:0 (n = 7)

Figure S3: Putative structures of the annotated ceramide and glucosylceramide molecular species (1–64). [a]Only in extracts; [b]Only in fractions; [c]Exact position of the substituents and stereochemistry not known

Table S2: UHPLC-VIP-HESI-MS/MS characterization of ceramides and glucosylceramides in apple pomace

No	Compound	$[M+H]^+$ measured	$[M+H]^+$ calculated	$\Delta m/z$(ppm)	Formula	Rt (min)	CCS (Å^2)
1	GlcCer-d18:2 h16:0	714.5509	714.5515	-0.840	$C_{40}H_{76}NO_9$	4.98	284.1
2	GlcCer-t18:1 h22:0	816.656	816.6559	+0.122	$C_{46}H_{90}NO_{10}$	6.48	303.4
3	GlcCer-t18:1 h23:0	830.6709	830.6716	-0.843	$C_{47}H_{92}NO_{10}$	6.67	306.9
4	GlcCer-t18:1 h24:0[b]	844.6877	844.6872	+0.592	$C_{48}H_{94}NO_{10}$	6.69	309.7
5	Cer-d18:0 h16:0	556.5295	556.5299	-0.719	$C_{34}H_{70}NO_4$	5.71	261.3
6	Cer-d18:0 h22:0[b]	640.6228	640.6238	-1.561	$C_{40}H_{82}NO_4$	7.46	280.5
7	Cer-d18:0 h26:0	696.6855	696.6864	-1.292	$C_{44}H_{90}NO_4$	7.60	293.3
8	Cer-d18:1 h16:0[b]	554.5134	554.5143	-1.623	$C_{34}H_{68}NO_4$	5.39	256.2
9	Cer-d18:1 h22:0[a]	638.6074	638.6082	-1.253	$C_{40}H_{80}NO_4$	6.89	275.8
10	Cer-d18:1 h24:0	666.6381	666.6395	-2.100	$C_{42}H_{84}NO_4$	6.29	284.5
11	Cer-d18:2 h16:0	552.4982	552.4986	-0.724	$C_{34}H_{66}NO_4$	4.98	257.7
12	Cer-d18:2 h22:0	636.5911	636.5925	-2.199	$C_{40}H_{78}NO_4$	6.90	276.0
13	Cer-d18:2 h24:0	664.6226	664.6238	-1.806	$C_{42}H_{82}NO_4$	7.13	278.1
14	Cer-d18:2 h25:0[a]	678.6403	678.6395	+1.179	$C_{43}H_{84}NO_4$	7.36	284.5
15	Cer-d18:2 h26:0[a]	692.6546	692.6551	-0.722	$C_{44}H_{86}NO_4$	7.19	292.4
16	Cer-d18:3 h16:0[b]	550.4881	550.488	+0.182	$C_{34}H_{64}NO_4$	3.53	256.9
17	Cer-10-hydroxy-d18:2 h16:0 [b c]	566.4774	566.4779	-0.883	$C_{34}H_{64}NO_5$	3.63	260.5
18	Cer-10-hydroxy-d18:2 h24:0 [c]	680.6176	680.6188	-1.763	$C_{42}H_{82}NO_5$	6.38	285.3
19	Cer-t18:0 h16:0	572.5234	572.5249	-2.620	$C_{34}H_{70}NO_5$	5.50	265.9
20	Cer-t18:0 h21:0	642.6019	642.6031	-1.867	$C_{39}H_{80}NO_5$	6.77	281.6
21	Cer-t18:0 h22:0	656.6181	656.6188	-1.066	$C_{40}H_{82}NO_5$	6.94	284.0
22	Cer-t18:0 h23:0	670.6321	670.6344	-3.430	$C_{41}H_{84}NO_5$	7.11	287.5
23	Cer-t18:0 h24:0	684.6486	684.6501	-2.191	$C_{42}H_{86}NO_5$	7.25	289.9
24	Cer-t18:0 h25:0	698.6649	698.6657	-1.145	$C_{43}H_{88}NO_5$	7.86	287.3
25	Cer-t18:0 h26:0	712.6799	712.6814	-2.105	$C_{44}H_{90}NO_5$	7.50	296.4
26	Cer-t18:1 h16:0	570.5085	570.5092	-1.227	$C_{34}H_{68}NO_5$	5.16	261.3
27	Cer-t18:1 h20:0	626.571	626.5718	-1.277	$C_{38}H_{76}NO_5$	6.35	272.5
28	Cer-t18:1 h21:0[a]	640.5872	640.5875	-0.468	$C_{39}H_{78}NO_5$	6.57	275.7
29	Cer-t18:1 h22:0	654.6024	654.6031	-1.069	$C_{40}H_{80}NO_5$	6.47	278.4
30	Cer-t18:1 h23:0 [a]	668.6186	668.6188	-0.299	$C_{41}H_{82}NO_5$	6.86	277.9
31	Cer-t18:1 h24:0[b]	682.6336	682.6344	-1.172	$C_{42}H_{84}NO_5$	6.84	285.0
32	Cer-t18:1 h25:0	696.6491	696.6501	-1.435	$C_{43}H_{86}NO_5$	7.24	288.0
33	Cer-t18:1 h26:0	710.6626	710.6657	-4.362	$C_{44}H_{88}NO_5$	7.38	291.4
34	Cer-t18:1 h26:1[a]	708.6487	708.6501	-1.976	$C_{44}H_{86}NO_5$	7.57	285.5
35	GlcCer-d18:1 h16:0	716.5662	716.5671	-1.256	$C_{40}H_{78}NO_9$	5.41	285.8
36	GlcCer-d18:1 h22:0[b]	800.6604	800.661	-0.749	$C_{46}H_{90}NO_9$	6.60	301.8
37	GlcCer-d18:2 h14:0 [b]	686.5196	686.5202	-0.874	$C_{38}H_{72}NO_9$	3.21	265.4
38	GlcCer-d18:2 h15:0[b]	700.5353	700.5358	-0.714	$C_{39}H_{74}NO_9$	4.63	280.9
39	GlcCer-d18:2 h18:0	742.582	742.5828	-1.077	$C_{42}H_{80}NO_9$	5.65	290.8

40	GlcCer-d18:2 h22:0	798.6443	798.6454	-1.377	$C_{46}H_{88}NO_9$	6.62	302.5
41	GlcCer-d18:2 h24:0	826.6757	826.6767	-1.210	$C_{48}H_{92}NO_9$	6.96	307.1
42	GlcCer-d18:2 h26:0[b]	854.7072	854.708	-0.936	$C_{50}H_{96}NO_9$	7.27	313.0
43	GlcCer-d18:3 h16:0	712.5351	712.5358	-0.982	$C_{40}H_{74}NO_9$	4.47	284.5
44	GlcCer-d18:3 h22:0[b]	796.6287	796.6297	-1.255	$C_{46}H_{86}NO_9$	6.33	301.9
45	GlcCer-t18:1 h16:0	732.5611	732.562	-1.229	$C_{40}H_{78}NO_{10}$	4.71	288.5
46	GlcCer-t18:1h20:0	788.624	788.6246	-0.761	$C_{44}H_{86}NO_{10}$	6.02	298.4
47	GlcCer-t18:1 h21:0	802.6394	802.6403	-1.121	$C_{45}H_{88}NO_{10}$	6.25	301.2
48	GlcCer-t18:1 h22:1[b]	814.6391	814.6403	-1.473	$C_{46}H_{88}NO_{10}$	5.75	303.5
49	GlcCer-t18:1 h25:0	858.7014	858.7029	-1.747	$C_{49}H_{96}NO_{10}$	7.00	311.8
50	GlcCer-t18:1 h26:0	872.717	872.7185	-1.719	$C_{50}H_{98}NO_{10}$	7.14	314.6
51	GlcCer-10-hydroxy-d18:2 h14:0[b c]	702.5144	702.5151	-0.996	$C_{38}H_{72}NO_{10}$	6.01	272.6
52	GlcCer-10-hydroxy-d18:2 h16:0[b c]	730.5454	730.5464	-1.369	$C_{40}H_{76}NO_{10}$	3.43	286.8
53	GlcCer-10-hydroxy-d18:2 h24:0	842.6705	842.6716	-1.305	$C_{48}H_{92}NO_{10}$	6.84	309.7
54	GlcCer-10-hydroxy-d18:2 h25:0[c]	856.6856	856.6872	-1.868	$C_{49}H_{94}NO_{10}$	6.33	311.2
55	GlcCer-t18:0 h24:0	846.7017	846.7029	-1.417	$C_{48}H_{96}NO_{10}$	7.03	313.7
56	GlcCer-7,10-dihydroxy-d18:2 h16:0[c]	746.5434	746.5413	+2.813	$C_{40}H_{76}NO_{11}$	3.45	290.3
57	GlcCer-7,10-dihydroxy-d18:2 h24:0[c]	858.665	858.6665	-1.747	$C_{48}H_{92}NO_{11}$	6.08	312.1
58	GlcCer-7,10-dihydroxy-8-oxo-d18:0 h22:0[c]	848.6449	848.6458	-1.061	$C_{46}H_{90}NO_{12}$	5.43	310.7
59	GlcCer-7,10-dihydroxy-8-oxo-d18:0 h:24:0[c]	876.6761	876.6771	-1.141	$C_{48}H_{94}NO_{12}$	6.01	318.2
60	GlcCer-t18:3h22:0[b]	812.6235	812.6246	-1.354	$C_{46}H_{86}NO_{10}$	6.35	310.1
61	GlcCer-t18:3 h24:0[b]	840.6551	840.6559	-0.952	$C_{48}H_{90}NO_{10}$	6.14	315.1
62	GlcCer-8-oxo-d18:2 h16:0[b c]	728.5297	728.5307	-1.373	$C_{40}H_{74}NO_{10}$	3.45	292.0
63	GlcCer-10-hydroxy-8-oxo-t18:0 h23:0[c]	862.6606	862.6614	-0.927	$C_{47}H_{91}NO_{12}$	5.73	315.5
64	GlcCer-10-hydroxy-8-oxo-t18:1 h16:0[b c]	762.5353	762.5362	-1.180	$C_{40}H_{76}NO_{12}$	4.30	289.0

[a]Only in extracts; [b]Only in fractions; [c]Exact position of the substituents, double bonds and stereochemistry not known

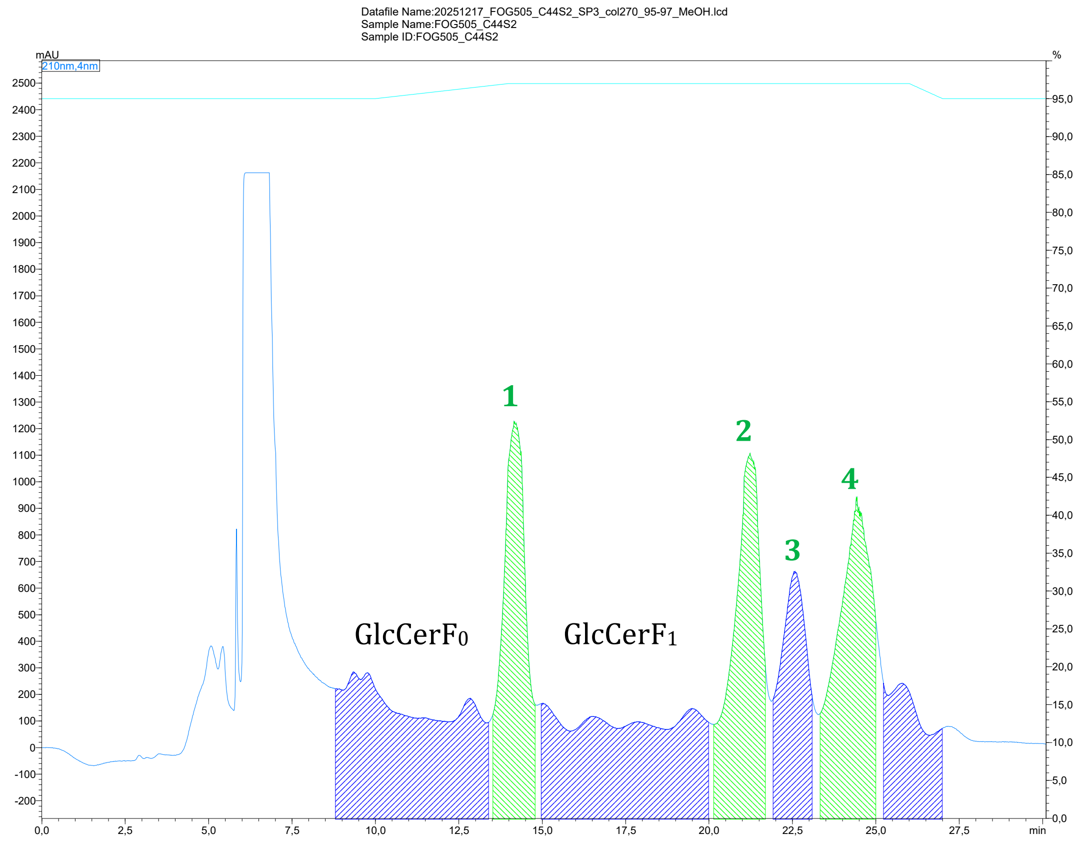

Figure S4: Semi-preparative HPLC chromatogram of the GlcCer-enriched precipitate showing the isolation of compounds 1–4 and the enriched fractions $GlcCerF_0$ and $GlcCerF_1$.

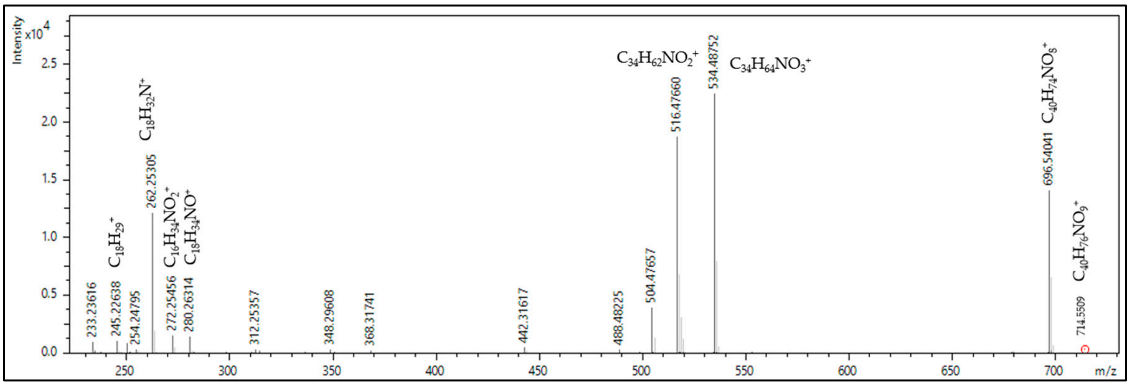

Figure S5: Suggested VIP-HESI/MS fragmentation pattern of GlcCer 1. Ion was extracted as extracted ions $[M+H]^+$, compound numbers as given in Table S2.

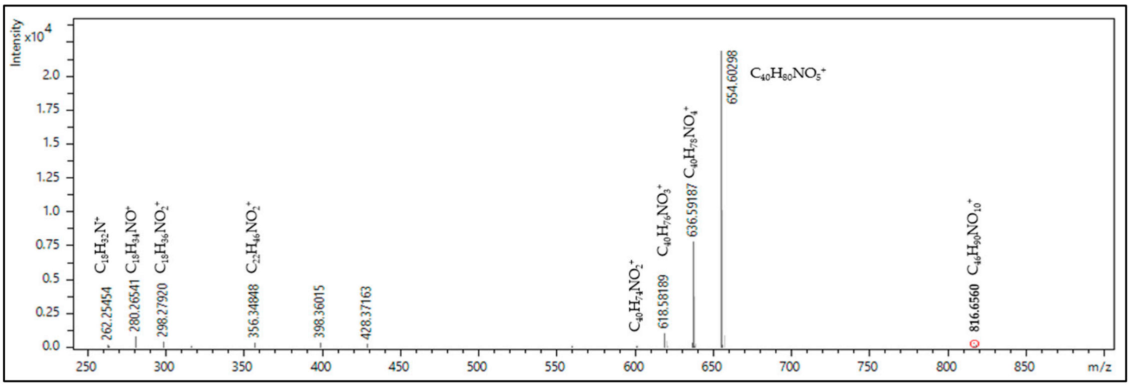

Figure S6: Suggested VIP-HESI/MS fragmentation pattern of GlcCer 2. Ion was extracted as extracted ions $[M+H]^+$, compound numbers as given in Table S2.

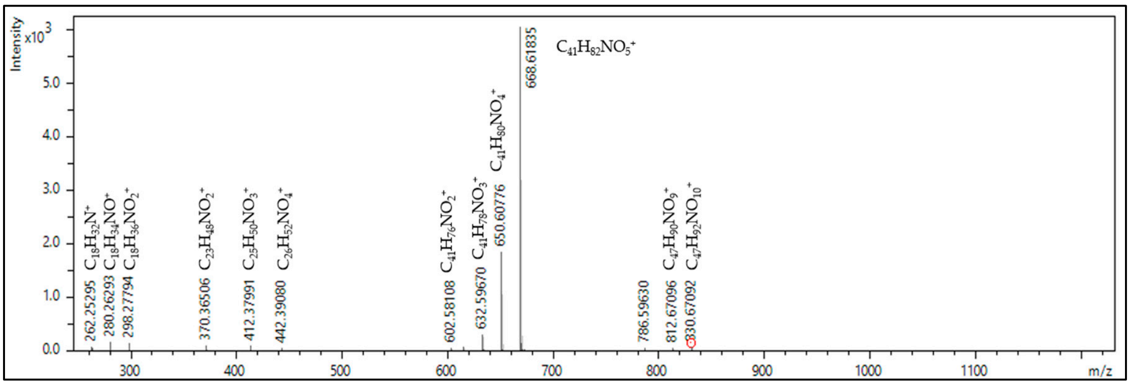

Figure S7: Suggested VIP-HESI/MS fragmentation pattern of GlcCer 3. Ion was extracted as extracted ions $[M+H]^+$, compound numbers as given in Table S2.

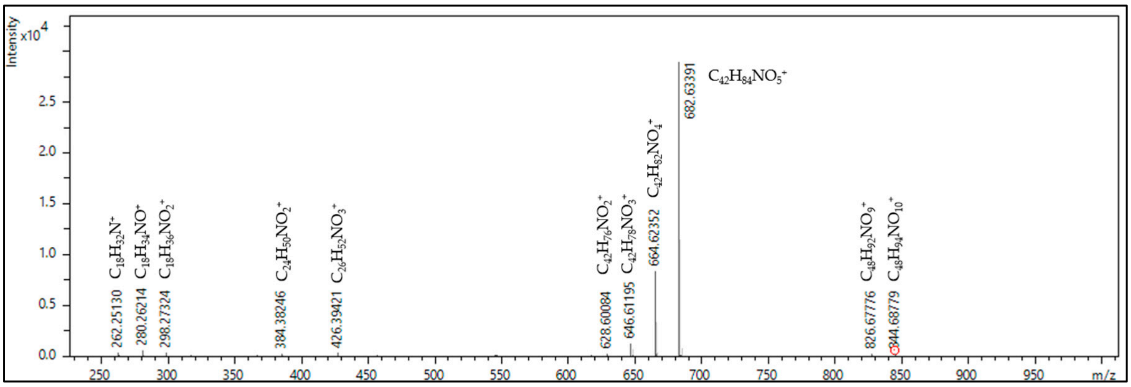

Figure S8: Suggested VIP-HESI/MS fragmentation pattern of GlcCer 4. Ion was extracted as extracted ions $[M+H]^+$, compound numbers as given in Table S2.

Compound 1

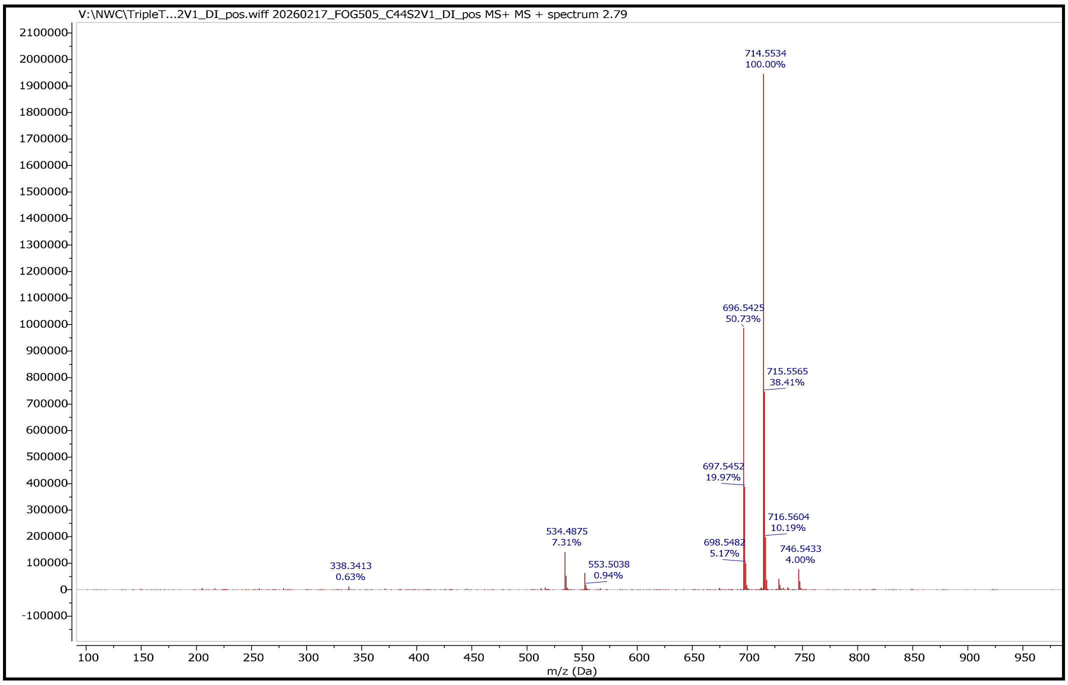

Figure S9: HRESIMS of compound 1

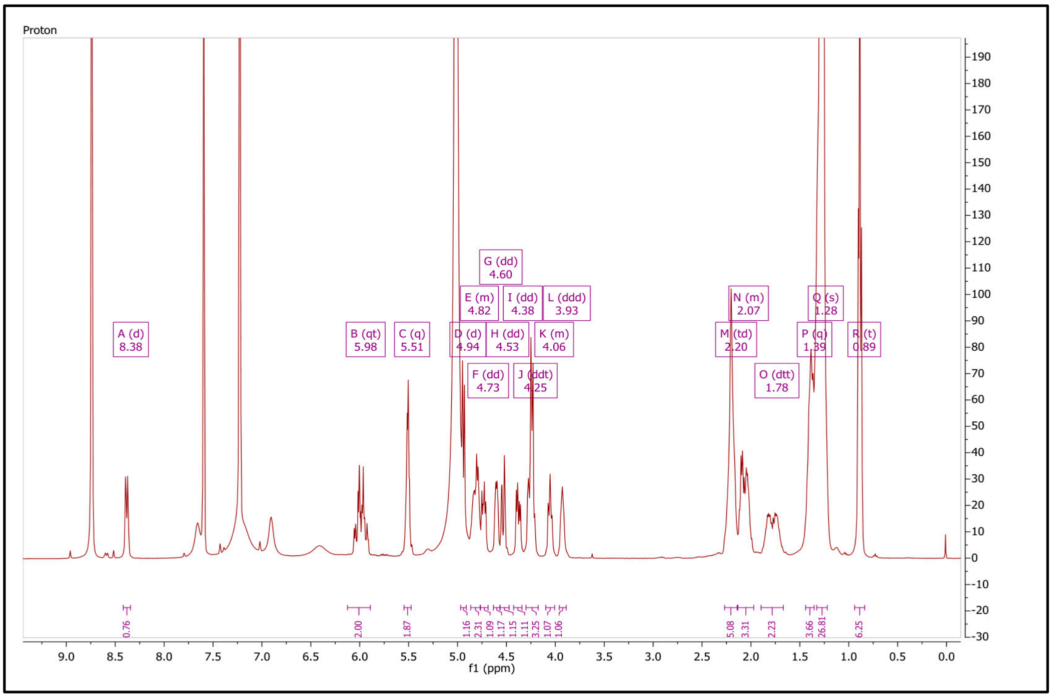

Figure S10: ^{1}H-NMR spectrum (Pyridine-d_5, 400 MHz) of compound 1

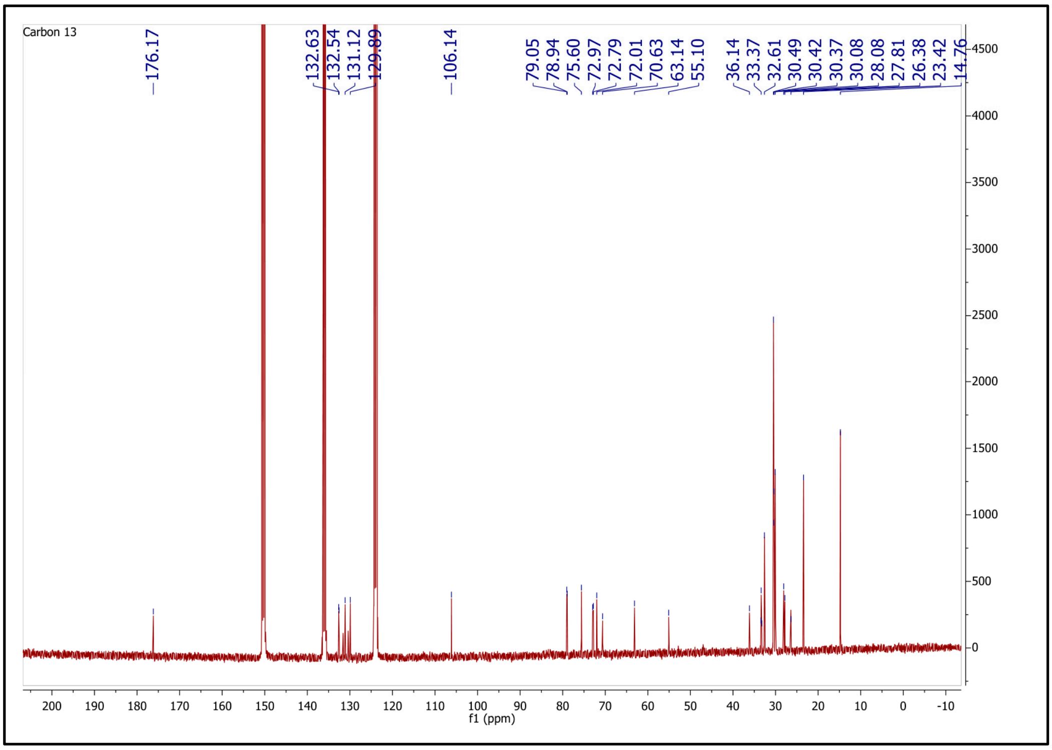

Figure S11: ^{13}C-NMR spectrum (Pyridine-d_5, 100 MHz) of compound 1

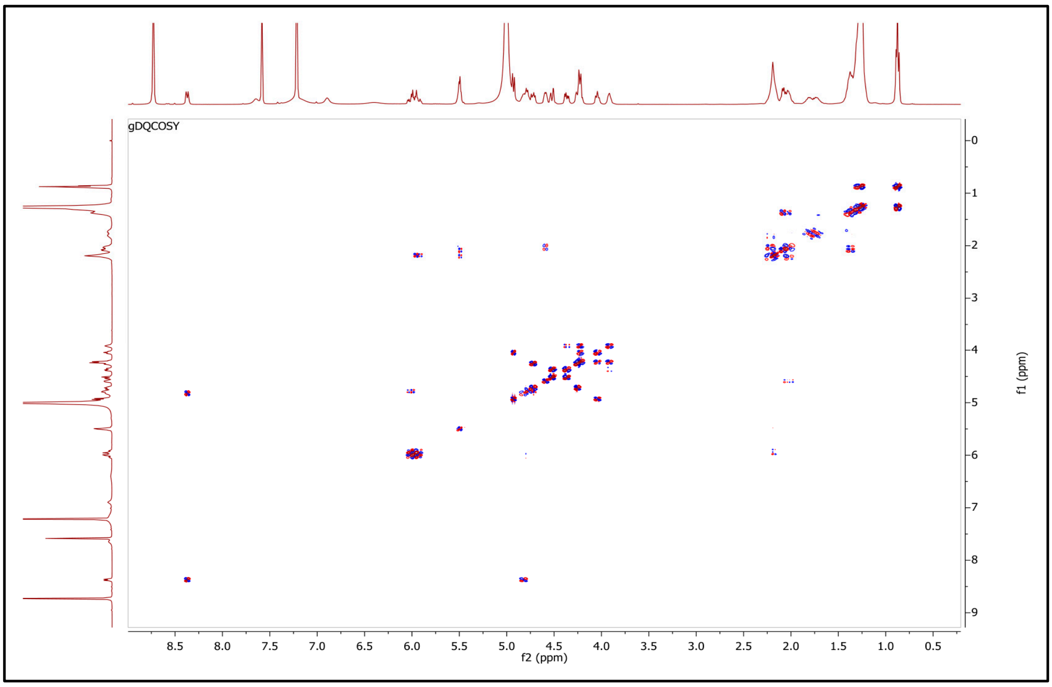

Figure S12: ^{1}H-^{1}H gDQCOSY spectrum of compound 1

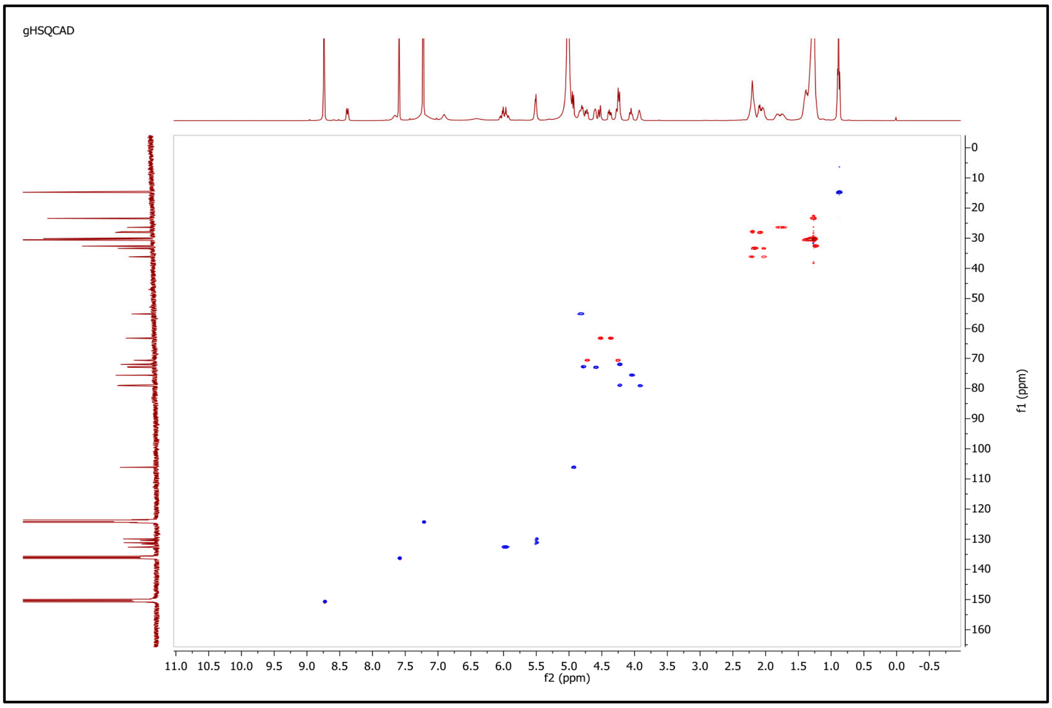

Figure S13: $^{1}H,^{13}C$ gHSQCAD spectrum of compound 1

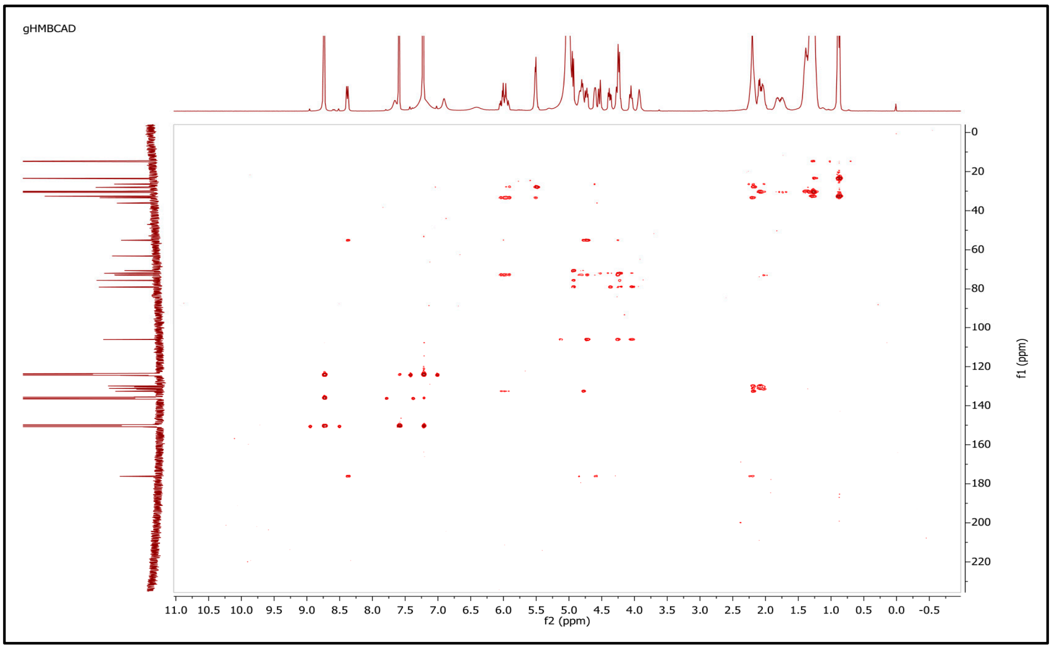

Figure S14: $^{1}H,^{13}C$ gHMBCAD spectrum of compound 1

Compound 2

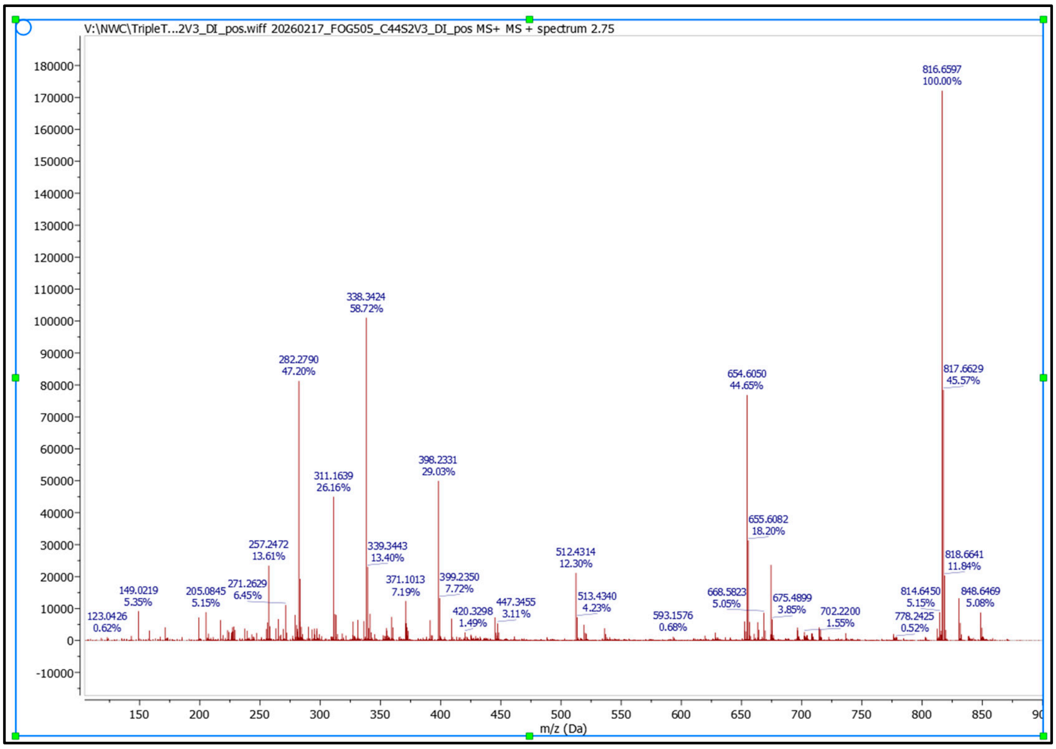

Figure S15: HRESIMS of compound 2

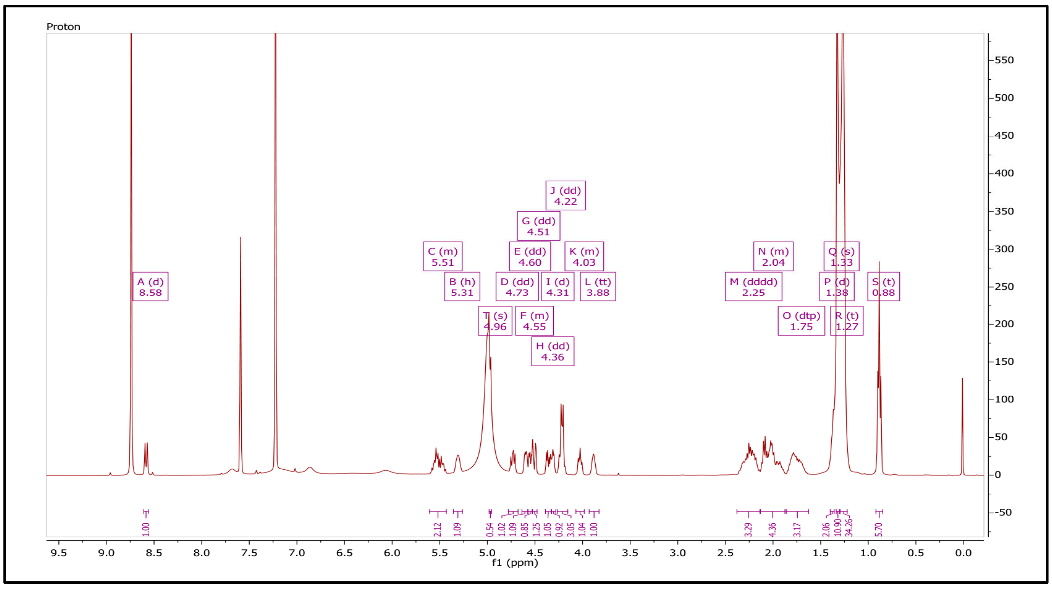

Figure S16: ^{1}H-NMR spectrum (Pyridine-d_5, 400 MHz) of compound 2

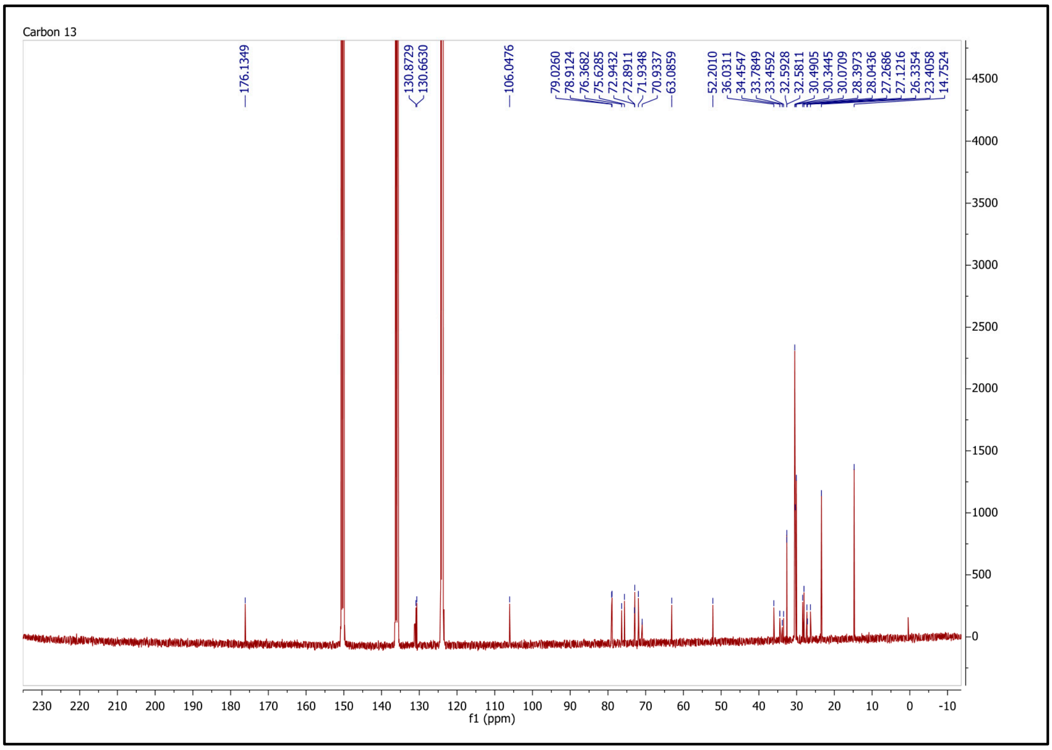

Figure S17: ^{13}C-NMR spectrum (Pyridine-d_5, 100 MHz) of compound 2

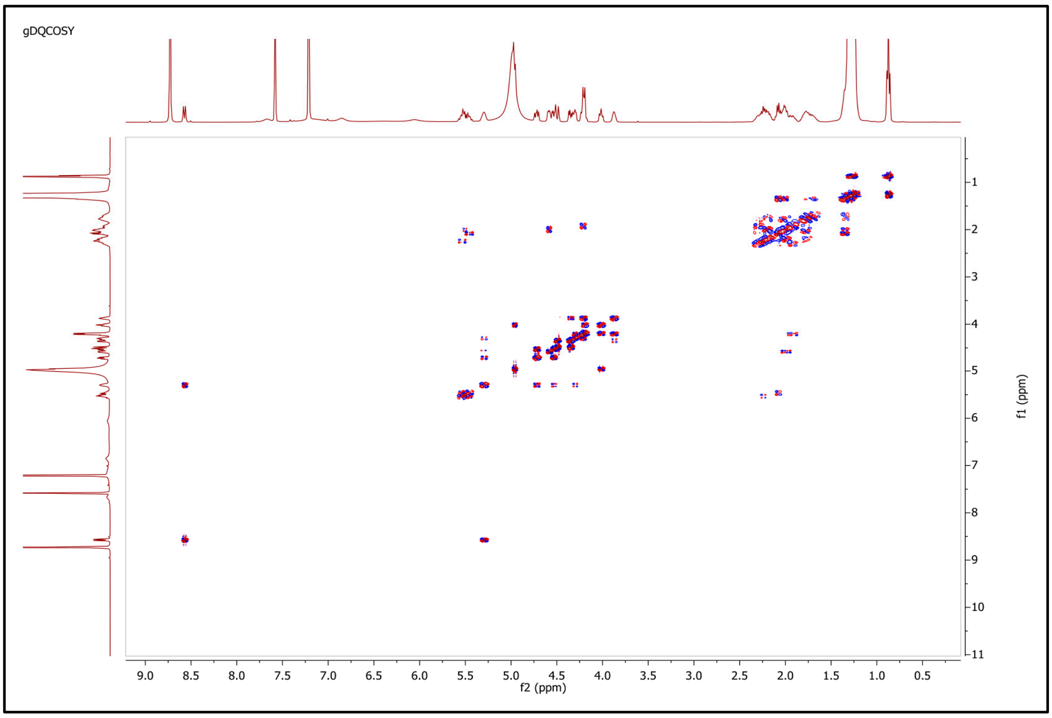

Figure S18: ^{1}H-^{1}H gDQCOSY spectrum of compound 2

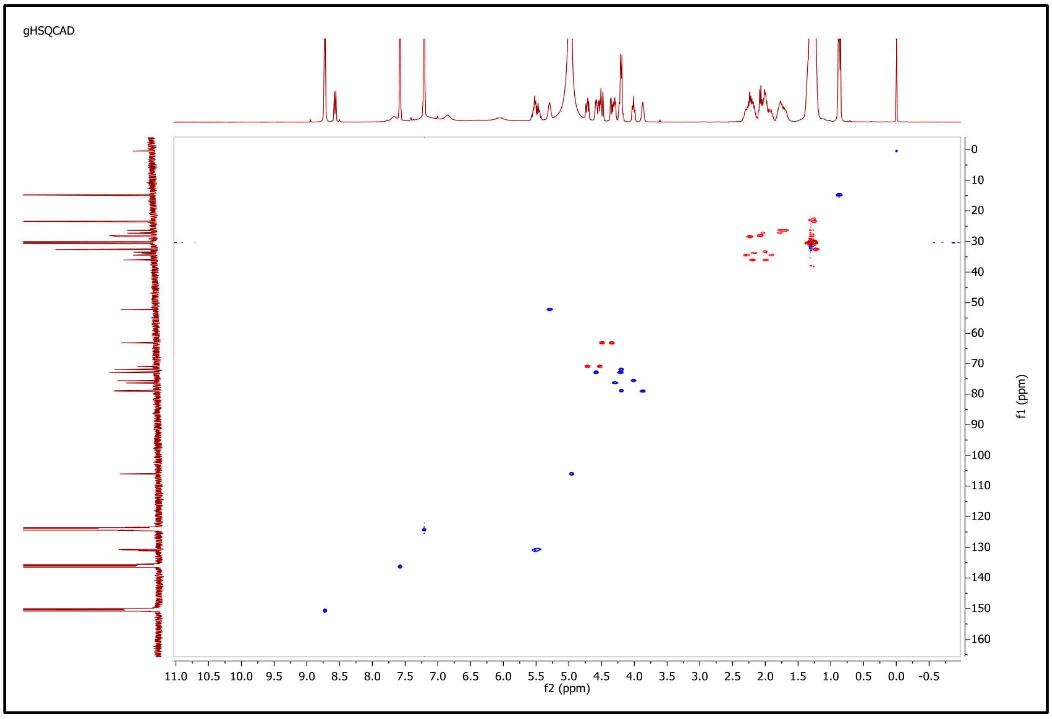

Figure S19: $^{1}H,^{13}C$ gHSQCAD spectrum of compound 2

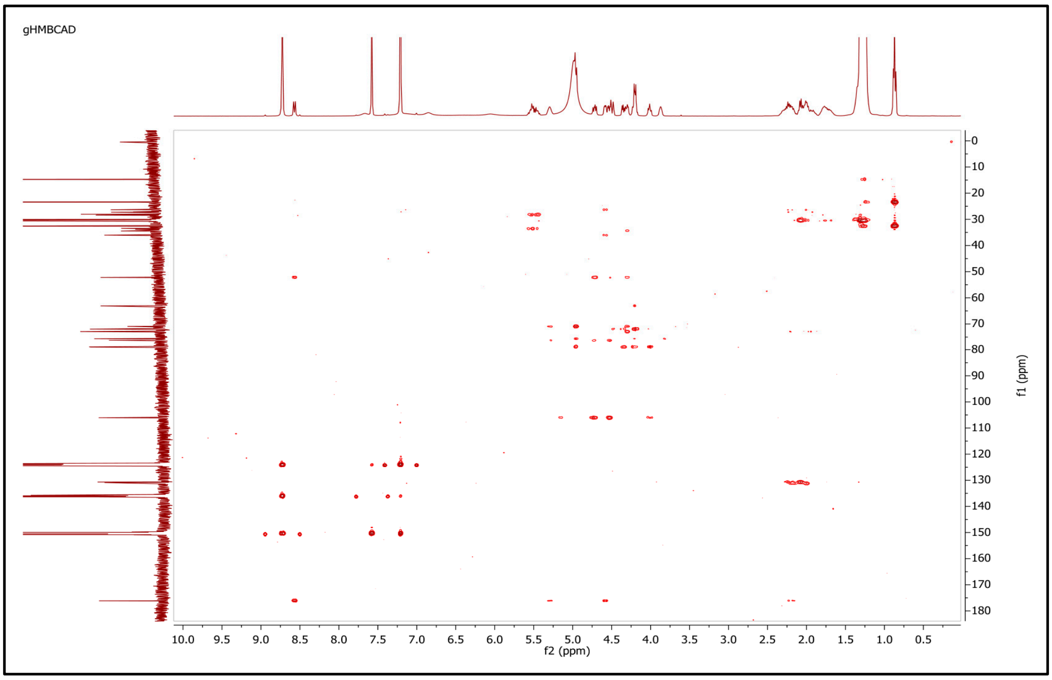

Figure S20: $^{1}H,^{13}C$ gHMBCAD spectrum of compound 2

Compound 3

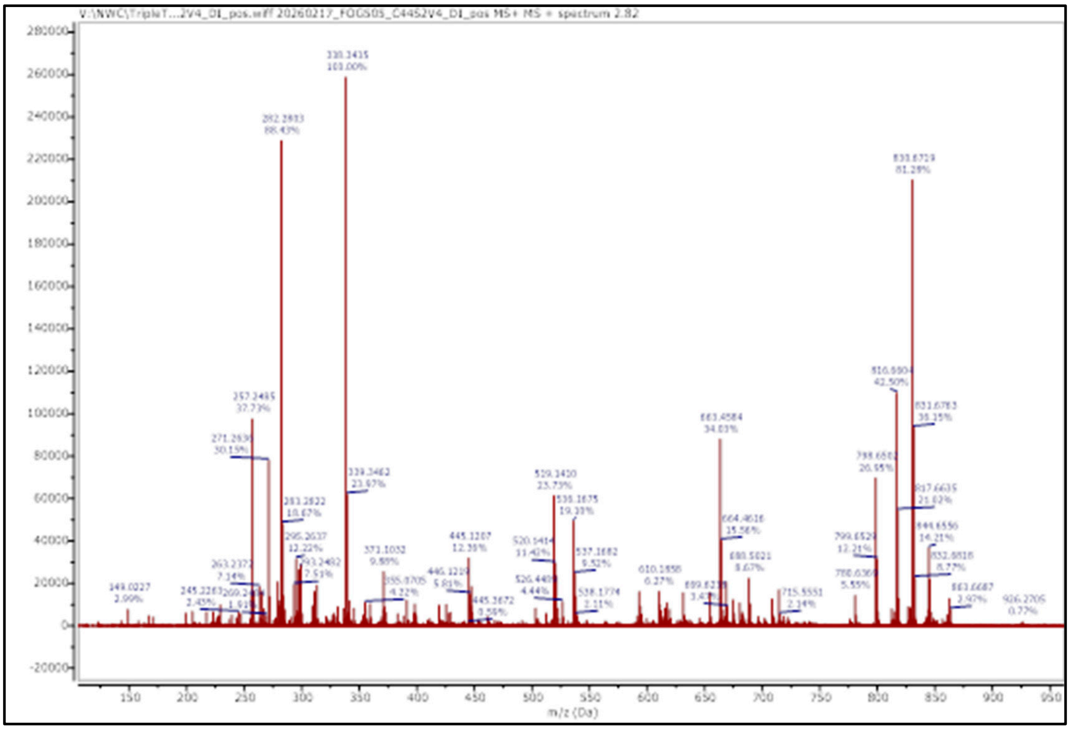

Figure S21: HRESIMS of compound 3

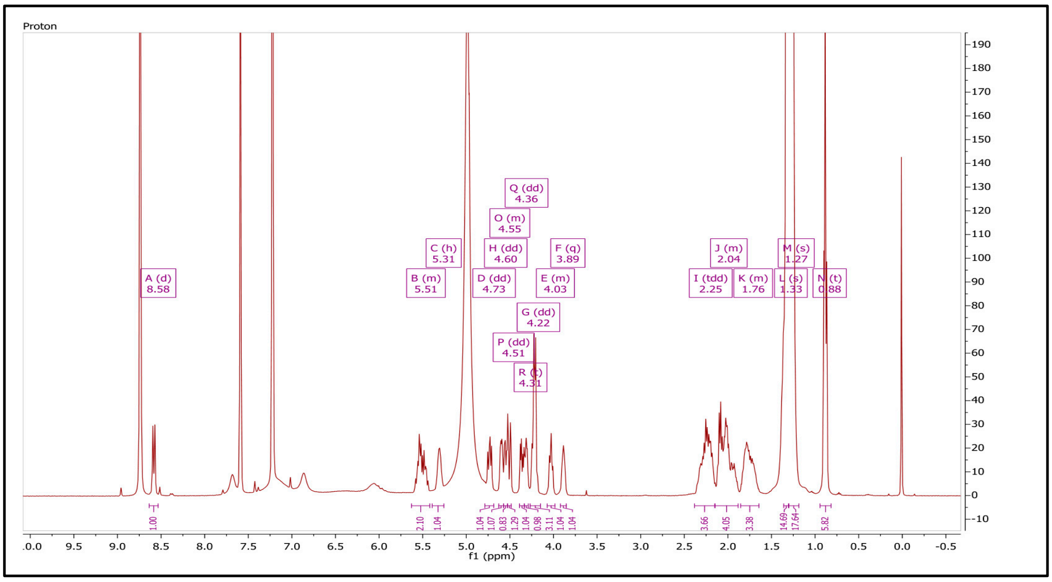

Figure S22: ^{1}H-NMR spectrum (Pyridine-d_5, 400 MHz) of compound 3

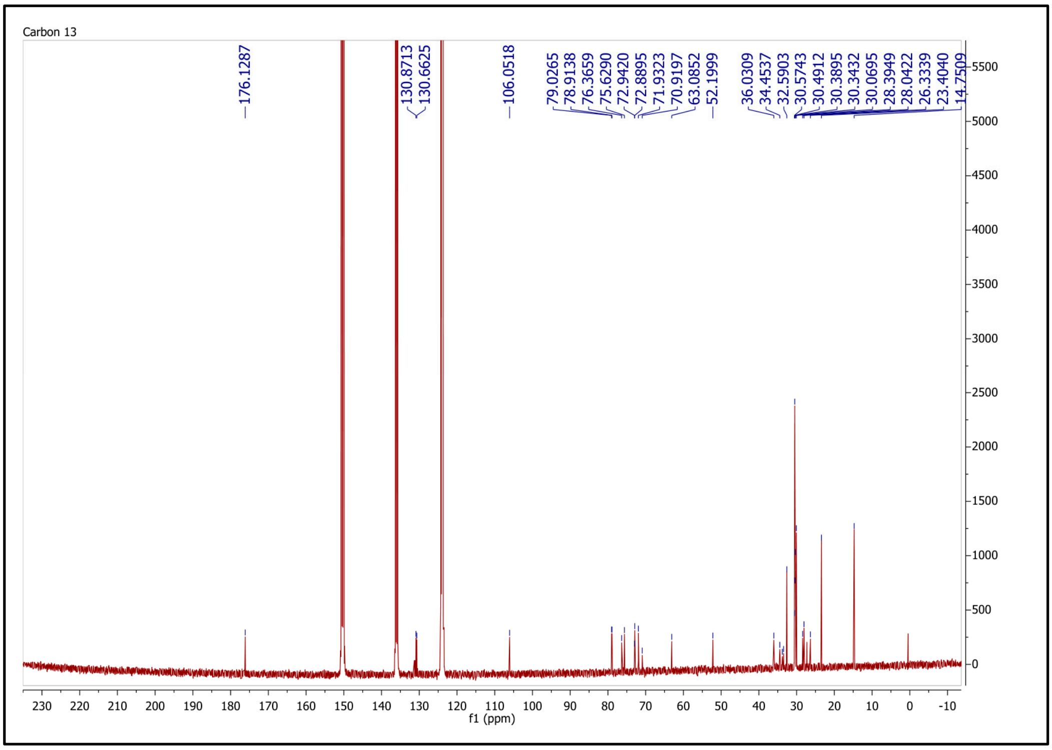

Figure S23: ^{13}C-NMR spectrum (Pyridine-d_5, 100 MHz) of compound 3

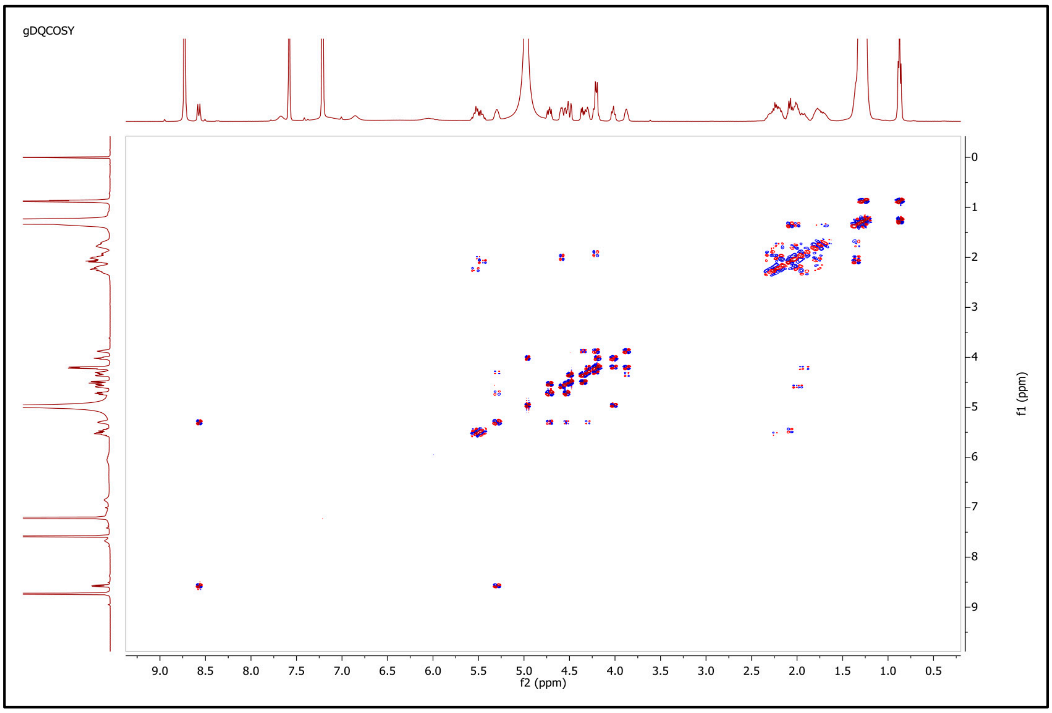

Figure S24: ^{1}H-^{1}H gDQCOSY spectrum of compound 3

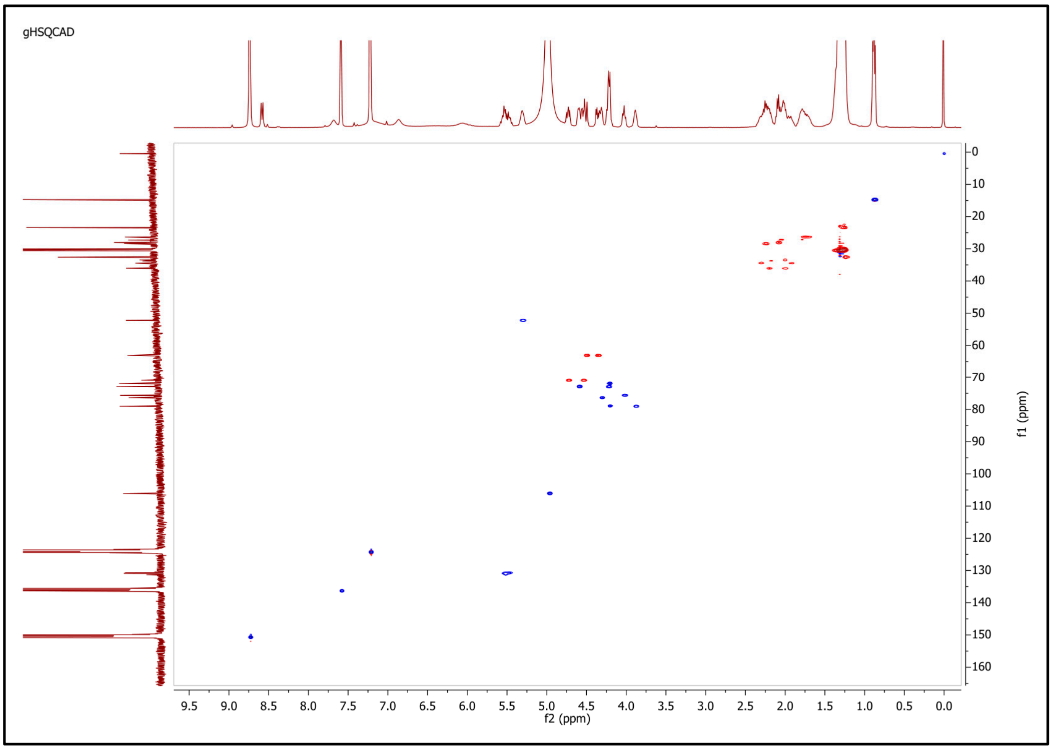

Figure S25: ^{1}H,^{13}C gHSQCAD spectrum of compound 3

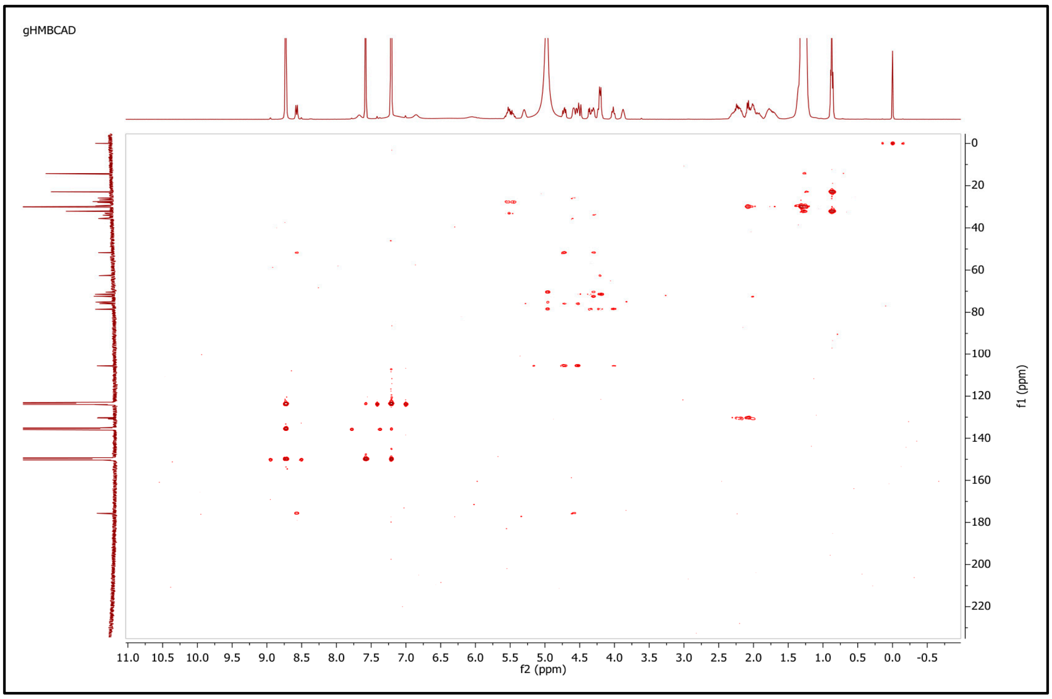

Figure S26: ^{1}H,^{13}C gHMBCAD spectrum of compound 3

Compound 4

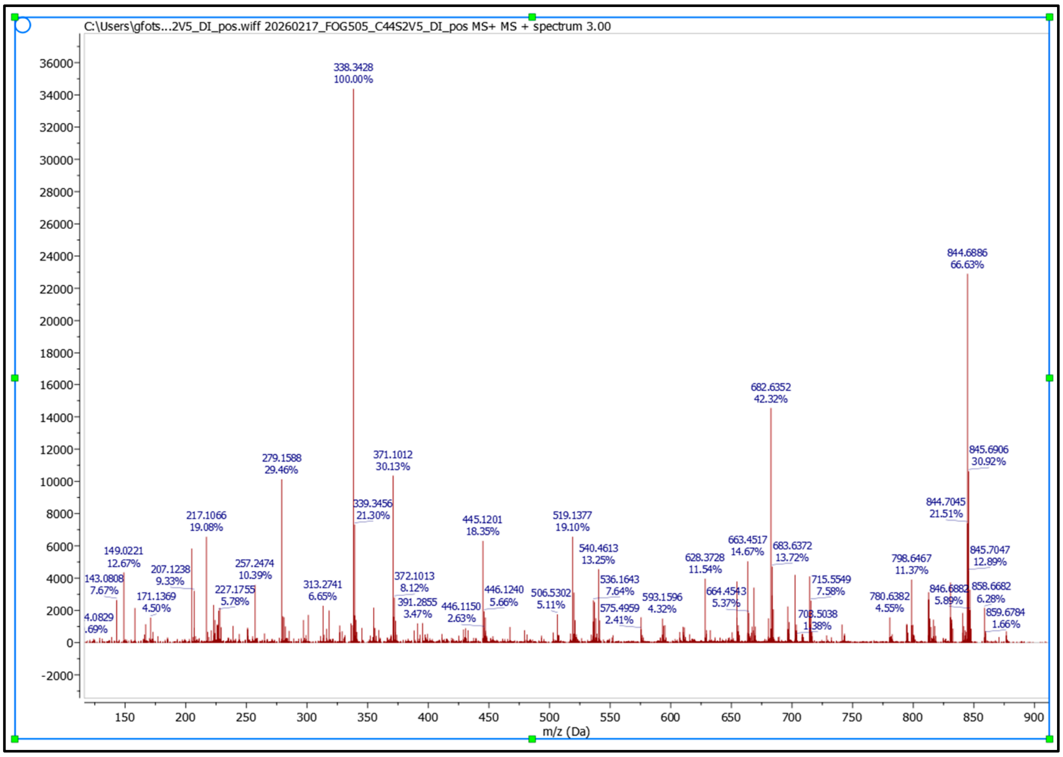

Figure S27: HRESIMS of compound 4

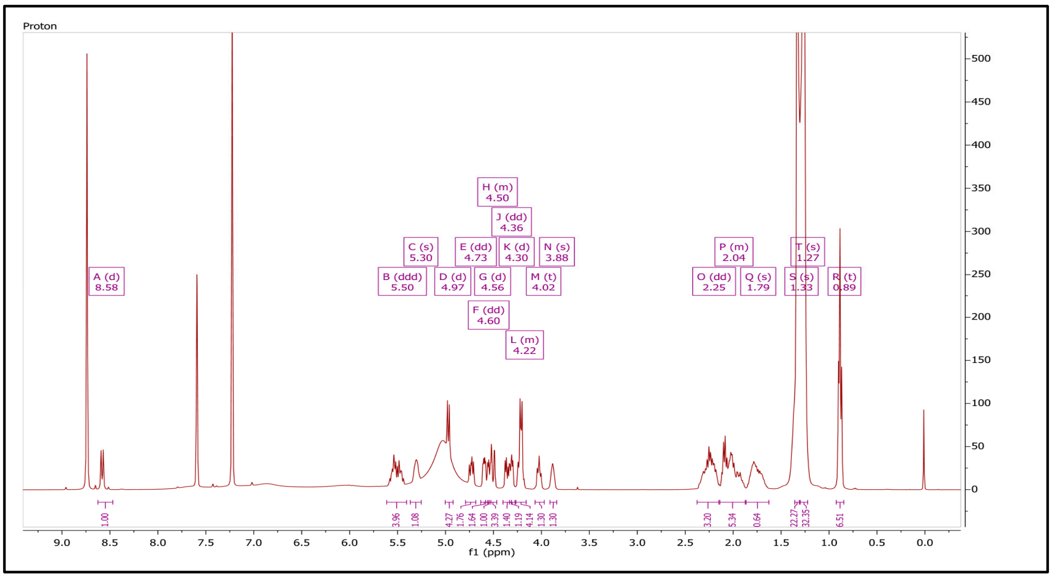

Figure S28: ^{1}H-NMR spectrum (Pyridine-d_5, 400 MHz) of compound 4

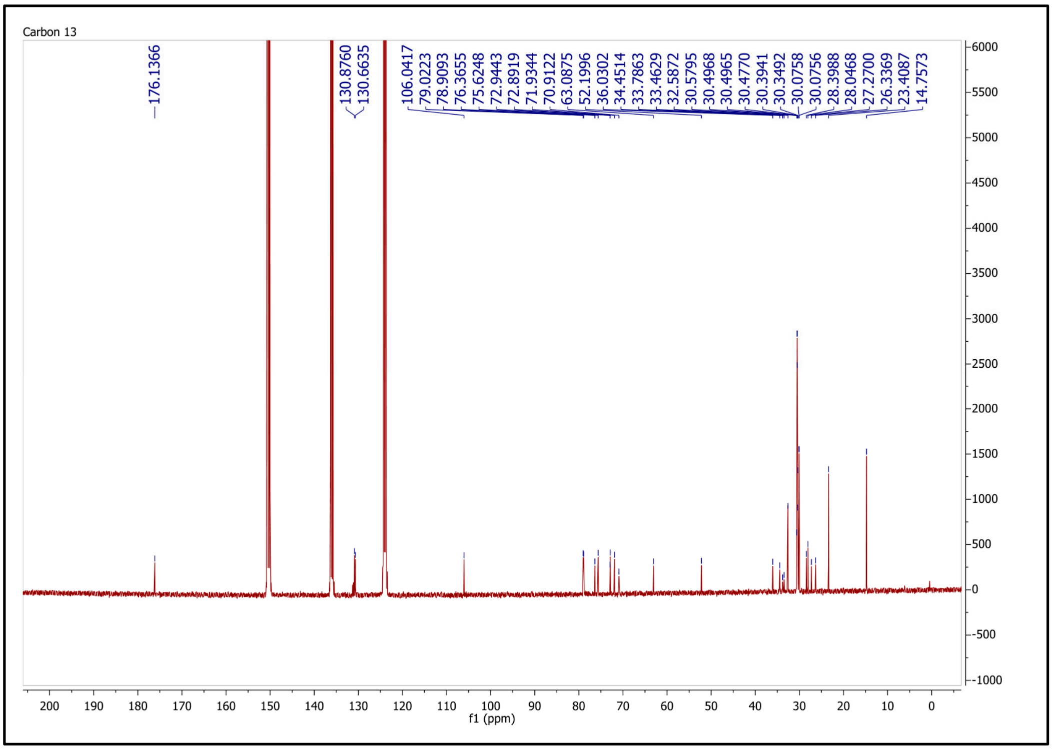

Figure S29: ^{13}C-NMR spectrum (Pyridine-d_5, 100 MHz) of compound 4

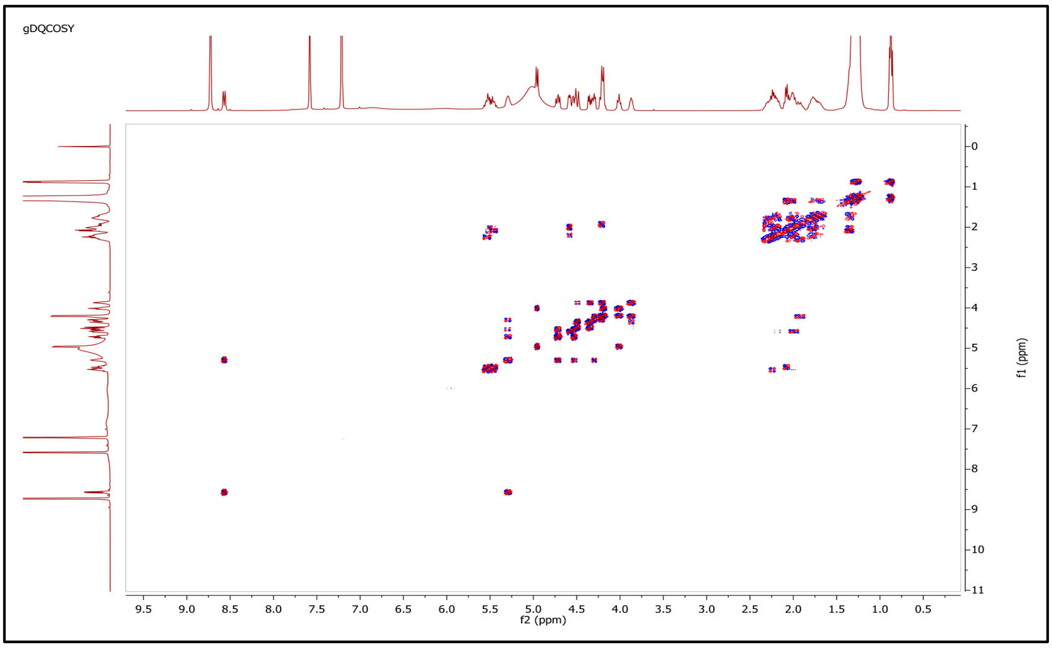

Figure S30: ^{1}H-^{1}H gDQCOSY spectrum of compound 4

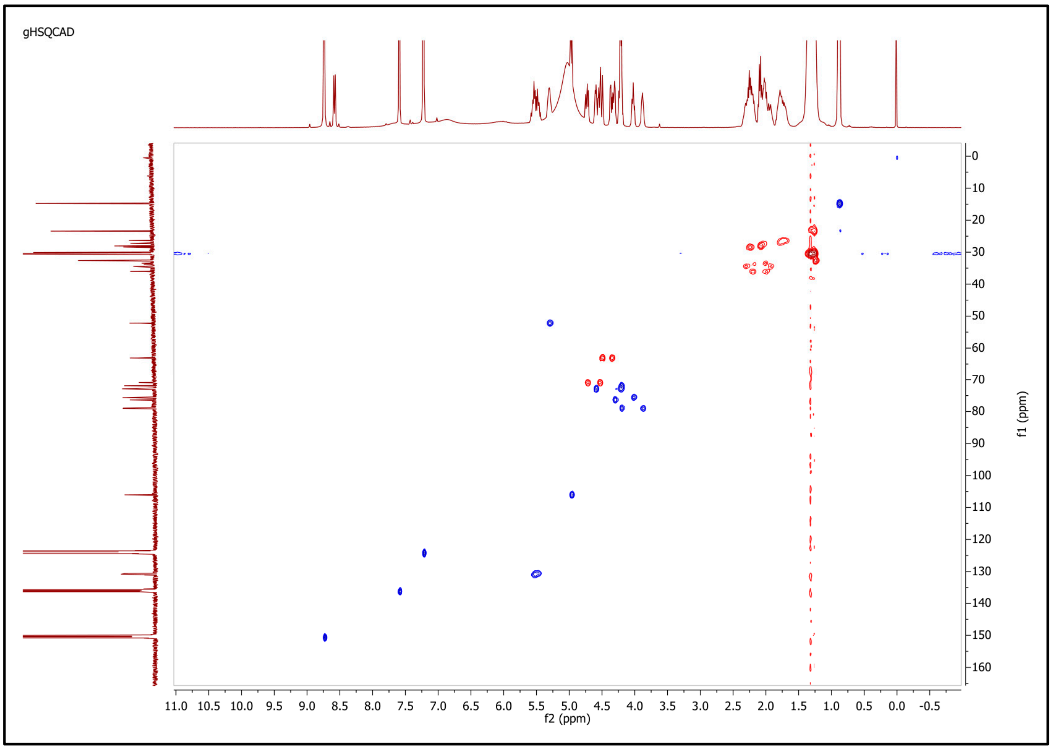

Figure S31: $^{1}H,^{13}C$ gHSQCAD spectrum of compound 4

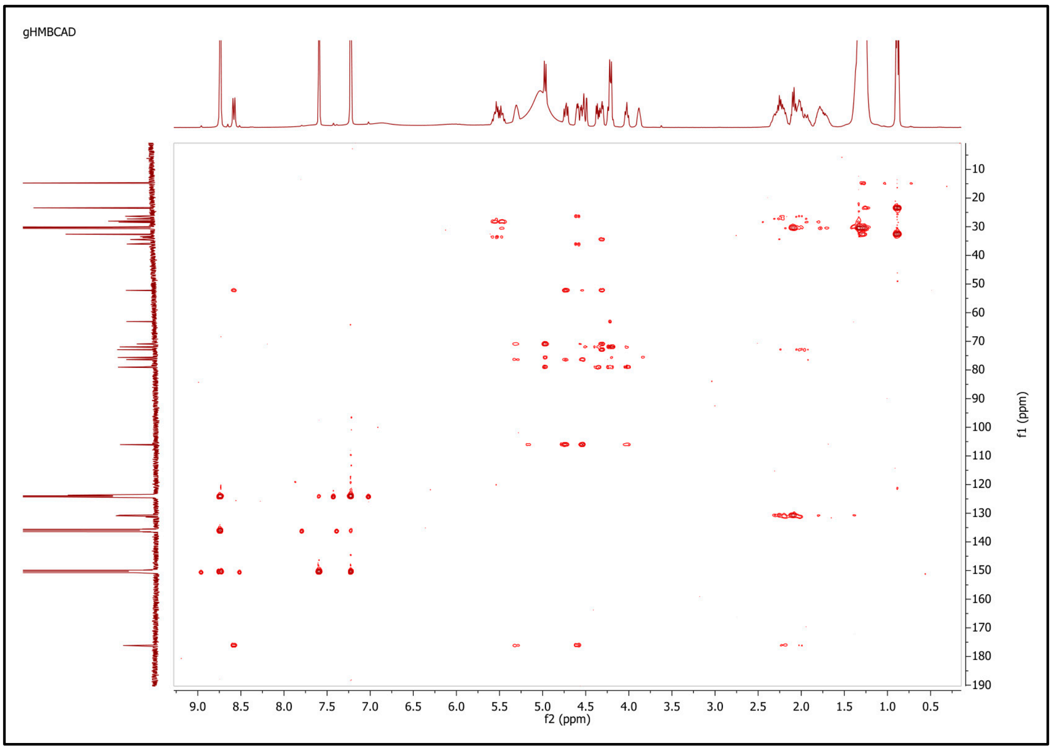

Figure S32: $^{1}H,^{13}C$ gHMBCAD spectrum of compound 4

Table S3: ^{1}H-NMR data (400 MHz, Pyridine-d_5, δ in ppm) of compounds 1-4

Pos.	1	2	3	4	HMBC
	δ_H (mult., *J* in Hz)	δ_H (mult., *J* in Hz)	δ_H (mult., *J* in Hz)	δ_H (mult., *J* in Hz)	
			**Sphingoid base**		
**-NH**	8.38 (1H, d, 8.5)	8.58 (1H, d, 9.1)	8.58 (1H, d, 9.1)	8.58 (1H, d, 9.2)	C-1, C-2, C-1′
**1**	4.24 (1H)*<br>4.73 (1H, dd,10.6, 5.8)	4.55* (1H)<br>4.73 (1H, dd, 10.7, 6.7)	4.53* (1H)<br>4.73 (1H, dd, 10.7, 6.6)	4.50* (1H)<br>4.73 (1H, dd, 10.5, 6.7)	C-2, C-3
**2**	4.82 (1H, m)	5.31 (1H, m)	5.31 (1H, m)	5.30 (1H, m)	C-1, C-3, C-1′
**3**	4.78 (1H, m)	4.31 (1H, d, 5.4)	4.31 (1H, d, 5.4)	4.30 (1H, d, 5.4)	C-1, C-2, C-4
**4**	5.98 (2H dt, 15.5, 5.5)	4.21* (1H)	4.21* (1H)	4.20* (1H)	C-2, C-3, C-5, C-6
**5**		1.90 (1H, m)<br>2.30 (1H, m)	1.91 (1H, m)<br>2.30 (1H, m)	1.91 (1H, m)<br>2.30 (1H, m)	C-3, C-4, C-6, C-7
**6**	2.19 (1H, m)	2.08 (2H, m)	2.08 (2H, m)	2.08 (2H, m)	
**7**	2.19 (2H, m)	2.19 (2H, m)	2.17 (2H, m)	2.17 (2H, m)	
**8**	5.51 (2H, m)	5.51 (2H, m)	5.51 (2H, m)	5.50 (2H, m)	C-6, C-7, C-9, C-10
**9**					
**10**	2.02 (1H, m)	2.00 (2H, m)	2.00 (2H, m)	2.00 (2H, m)	C-8, C-9, C-11
**11**	1.36 (m)				
**12–15**	1.28 (brs)	1.27 – 1.38 (brs)	1.27 – 1.33 (brs)	1.27 – 1.33 (brs)	
**16**	1.25*	1.23*	1.24*	1.24*	C-18
**17**	1.28*	1.25*	1.25*	1.25*	C-18
**18**	0.89 (3H, t, 6.6)	0.88 (3H, t, 6.6)	0.88 (3H, t, 6.6)	0.89 (3H, t, 6.5)	C-16, C-17
			**Fatty acid**		
**1′**	C=O	C=O	C=O	C=O	
**2′**	4.60 (1H, dd, 8.2, 3.7)	4.60 (1H, dd, 8.0, 3.7)	4.60 (1H, dd, 7.9, 3.7)	4.60 (1H, dd, 7.6, 3.4)	C-1′, C-3′, C-4′
**3′**	2.02 (1H, m)<br>2.22 (1H, m)	2.00 (1H, m)<br>2.20 (1H, m)	2.00 (1H, m)<br>2.20 (1H, m)	1.99 (1H, m)<br>2.19 (1H, m)	
**4′**	1.78 (2H, m)	1.73 (2H, m)	1.74 (2H, m)	1.73 (2H, m)	
**5′**	2.19 (m)	2.24 (m)	2.24 (m)	2.24 (m)	
**6′–n′**[a]	1.28 (br s)	1.27 – 1.38 (br s)	1.27 – 1.33 (br s)	1.27 – 1.33 (br s)	
**ω-2**	1.25*	1.23*	1.24*	1.24*	CH3
**ω-1**	1.28*	1.25*	1.25*	1.25*	CH3
**ω-CH3**	0.89 (3H, t, 6.6)	0.88 (3H, t, 6.6)	0.88 (3H, t, 6.6)	0.89 (3H, t, 6.5)	ω-1, ω-2
			**Gucoside**		
**1″**	4.94 (1H, d, 7.8)	4.96*	4.96 (1H)*	4.97 (1H, d, 7.7)	C-1
**2″**	4.06 (1H, m)	4.03 (1H, m)	4.03 (1H, m)	4.02 (1H, t, 8.0)	C-1″, C-3″, C-4″
**3″**	4.24 (1H)*	4.22 (1H, dd, 10.8, 6.3)	4.22 (1H, dd, 10.8, 6.3)	4.22*	C-1″, C-2″, C-4″, C-5″
**4″**	4.24 (1H)*	4.22 (1H)*	4.22 (1H)*	4.22 (1H)*	C-2″, C-3″, C-5″, C-6″
**5″**	3.93 (1H, ddd, 9.0, 5.4, 2.5)	3.88 (1H, m)	3.89 (1H, m)	3.88 (1H, brs)	C-3″, C-4″, C-6″
**6″**	4.38 (1H, dd, 11.8, 5.3)<br>4.53 (1H dd, 11.8, 5.3)	4.36 (1H, dd, 11.8, 5.3)<br>4.51 (1H, dd, 11.9, 2.6)	4.36 (1H, dd, 11.8, 5.4)<br>4.51 (1H, dd, 11.9, 2.6)	4.36 (1H, dd, 11.8, 5.3)<br>4.50 (1H, m)	C-4″, C-5″

[a] compound **1** (*n*′ = 13), compound **2** (*n*′ = 19), compound **3** (*n*′ = 20), compound **4** (*n*′ = 21)
